# Gamma-aminobutyric acid treatment promotes resistance against *Sogatella furcifera* in rice

**DOI:** 10.3389/fpls.2024.1419999

**Published:** 2024-07-18

**Authors:** Rahmatullah Jan, Saleem Asif, Sajjad Asaf, Lubna, Zakirullah Khan, Waleed Khan, Kyung-Min Kim

**Affiliations:** ^1^ Department of Applied Biosciences, Graduate School, Kyungpook National University, Daegu, Republic of Korea; ^2^ Coastal Agriculture Research Institute, Kyungpook National University, Daegu, Republic of Korea; ^3^ Natural and Medical Science Research Center, University of Nizwa, Nizwa, Oman

**Keywords:** antioxidant, gamma-aminobutyric acid, melatonin, phytohormone, *Sogatella furcifera*, tricarboxylic acid cycle

## Abstract

The *Sogatella furcifera* (Horváth) (Homoptera: Delphacidae) is a white-backed planthopper (WBPH) that causes “hopper burn” in rice, resulting in severe yield loss. Gamma-aminobutyric acid (GABA) is a well-known neurotransmitter that inhibits neurotransmission in insects by binding to specific receptors. In this study, we investigated the potential role of GABA in modulating rice resistance to WBPH and evaluated possible defense mechanisms. The experiment was conducted in green house in pots consist of four groups: control, GABA-treated, WBPH-infested, and WBPH-infested treated with GABA. Among the various tested concentration of GABA, 15 mM GABA was applied as a single treatment in water. The treatment was administered one week before WBPH infestation. The results revealed that 15 mM GABA treatment strongly increased WBPH resistance. A plate-based assay indicated that direct application of 15 mM GABA increased the mortality rate of WBPH and increased the damage recovery rate in rice plants. We found that GABA treatment increased the activation of antioxidant enzymes and reduced the reactive oxygen species content and malondialdehyde contents, and reduced the damage rate caused by WBPH. Interestingly, GABA-supplemented plants infested with WBPH exhibited increased phenylalanine ammonia-lyase and pathogenesis-related (PR) genes expression levels. GABA induced the accumulation of abscisic acid (ABA) and salicylic acid (SA) and enhanced the stomata closure and reduced leaf vessels to reduce water conductance during WBPH stress. Furthermore, we found that GABA application to the plant induced the expression of Jasmonic acid (JA) biosynthesis genes (*LOX*, *AOS*, *AOC*, and *OPR*) and melatonin biosynthesis-related genes (*TDC*, *T5H*, *ASMT*, and *SNAT*). Our study suggested that GABA increases resistance against WBPH infestation by regulating antioxidant defense system, TCA cycle regulation, phytohormonal signaling, and PR gene regulation.

## 1 Introduction

In Asia, the white-backed planthopper (*Sogatella furcifera*; WBPH) is the most abundant and detrimental pest found in rice fields (Horgan et al., 2020). This insect ingests plant cell sap, causing “hopper burn” and severely infests paddy fields and reduces yield (Suri and Singh, 2011). In plants, WBPH infestation causes dwarfism and is marked by a reduction in leaf area, dry matter, nitrogen concentration in stems and leaves, and photosynthesis rate (Prasad et al., 2010). WBPH can also indirectly damage rice by acting as a vector for viruses such as rice black streak dwarf virus-2 and southern rice black streak dwarf virus (Zhang et al., 2008; Zhou et al., 2008). The WBPH has emerged as a significant threat to rice yields, capable of causing considerable damage and reducing crop yield significantly, with reported losses in Japan ranging from 10 to 90% depending on the severity of infestation (Khatri et al., 1983). It has been reported in 1983 that the infestation of WBPH at levels ranging from 15 to 200 insects per rice hill resulted in percentage losses of paddy rice ranging from 11-37% across different growth stages and exposure periods, with the lowest population level of 15 insects per hill causing 13-37% loss depending on the growth stage and duration of exposure (Khatri et al., 1983). In South Korea, WBPH migrates from Southern China between the end of June and beginning of July, when rice is at the seedling stage and most susceptible (Kim et al., 2021). This pest induces stress in plants through generation of reactive oxygen species (ROS), which leads to cellular damage, programmed cell death, and reduced plant yield.

Gamma-aminobutyric acid (GABA) is a non-proteinogenic amino acid found in all plants. It is a four-carbon amino acid synthesized by decarboxylation of glutamate in the cytosol and plastid and plays an important role in plant growth and development (Scholz et al., 2015; Jalil et al., 2019; Du et al., 2020). It synthesized from glutamate through a series of reactions (GABA shunt), catalyzed by glutamate decarboxylase (GAD) followed by conversion to succinate through two reactions catalyzed by GABA transaminase (GABA-T) and succinic semialdehyde dehydrogenase (SSADH) (Khan et al., 2021b). Succinic acid is not involved directly in stress resilience, however studies have shown that it is involved in TCA cycle and enhance plant energy during environmental stress. It is reported that exogenous GABA enhanced indigenous GABA level which is metabolize to succinic acid and fed into the TCA cycle (Hijaz and Killiny, 2019). GABA is involved in plant defense systems against both abiotic and biotic stress. Commonly, plants induce calcium ion (Ca^2+^) production in response to stress, resulting in the formation of a Ca^2+^/calmodulin complex. This complex is recognized by GAD in the cytosol, triggering accumulation of GABA. GABA can then enter into the tricarboxylic acid (TCA) cycle and maintain carbon and nitrogen equilibrium, or it can inhibit ROS generation via activation of antioxidant enzymes (Li et al., 2021b). GABA may also act as a signaling molecule for activation of biomolecules in plants against various stresses. Briefly, abiotic and biotic stress induces GABA accumulation in plants, which enhance tolerance to stress.

The stresses that induce GABA accumulation are; low O_2_, low and high temperature, drought stress, salt stress, heavy metal stress, pests infestation, bacteria, and fungi infection (Shelp et al., 2021). Therefore, some studies propose that GABA might control various pathways in cell metabolism and stress responses simultaneously however, these mechanisms are not fully understood yet (Kumar et al., 2017). Several studies have reported that both genetic manipulation of endogenous GABA and application of exogenous GABA modulate plant stress tolerance. For instance, tobacco and *Arabidopsis* with endogenously elevated GABA display enhanced tolerance to attack by *Agrobacterium*, *Pseudomonas*, insect larva, and root-knot nematode compared to wild plants (Lancien and Roberts, 2006; Eisenach et al., 2017; Van Kleeff et al., 2018; Saito and Uozumi, 2019; Kar et al., 2021). However, tomato plants with low GABA levels showed reduced tolerance to *Ralstonia* infection (Chen et al., 2013). A recent study also reported that *GAD* mutation, which reduces GABA, glutamine, and alanine levels in *Arabidopsis* resulted in *Pst* and *Pst-avrRpt2* susceptibility (Deng et al., 2020). Many studies have reported that GABA accumulates in plants during mechanical stimulation and tissue damage, which is likely a component of the plant defense system against herbivorous insects (Wallace et al., 1984; Ramputh and Bown, 1996; Bown et al., 2006; Huang et al., 2011; Mithöfer and Boland, 2012). GABA is known to target the nervous system of invertebrates; therefore, high concentrations could inhibit the neuronal transmission of insect nervous system and act as a defense tool against herbivorous insects (Huang et al., 2011; Tarkowski et al., 2020). It has been reported by Irving et al., 1979, that GABA inhibit the neuromuscular junction of the insects and causes insect paralysis (Irving et al., 1979). They injected different compounds including GABA into *Lucilia sericata* larva by using specialized Agla syringe fitted with a Gillette 26G hypodermic needle. However, there was a lack of specific concentrations for each compound mentioned. Nevertheless, it was noted that the effective concentration of the injected doses was sufficiently low to be physiologically relevant. Additionally, Casida and Durkin, 2015 explained that, GABA helps regulate muscle activity. When a pesticides act as GABA agonists, they mimic GABA and activate the Cl^-^ channels, causing an excessive flow of chloride ions. On the other hand, when a pesticides act as GABA antagonists, they block the Cl^-^channels, preventing chloride ions from moving. Both actions disrupt the normal muscle activity of the pests, which can lead to insect paralysis and death (Casida and Durkin, 2015). Exogenous application of GABA also increases endogenous GABA levels in plants and enhances tolerance to several abiotic stresses (Shelp et al., 2021). Plant generates ROS in the form of free radicle during stress condition which causes oxidative stress. Previous study demonstrated that GABA has the capability to scavenge the free radicle and reduce the ROS which results into reduced oxidative stress (Smirnoff and Cumbes, 1989). GABA on the other hand reduces ROS indirectly by enhancing antioxidant enzymes such as SOD, glutathione (GSH) and GPX. These enzymes also scavenge free radical and reduce oxidative stress. Another study demonstrated that increased endogenous GABA accumulation regulates non-enzymatic antioxidants (ascorbic acid, reduced glutathione, and phenol), enzymatic antioxidants (superoxide dismutase, ascorbate peroxidase, glutathione reductase, glutathione peroxidase, glutathione S-transferase, and catalase), and osmolytes including amino acids (Shelp et al., 2021). Furthermore, studies have shown that application of GABA to tomato and pear plants reduces biotic stress from fungal pathogens via induction of catalase and peroxidase antioxidant enzymes and inhibits plant cell death caused by ROS (Yu et al., 2014; Fu et al., 2017; Yang et al., 2017). Several studies have also reported that GABA increases nitric oxide, which is linked with the antioxidant defense system and regulation of gene expression (Kalhor et al., 2018; Tang et al., 2020; Ageeva-Kieferle et al., 2021). GABA induces stress tolerance through regulation of hormonal pathways, such as abscisic acid (ABA), salicylic acid (SA), and jasmonic acid (JA), which control expression of stress related genes and transcriptional factors (Renault et al., 2011; Scholz et al., 2015; Li et al., 2019; Podlešáková et al., 2019). Overall, the existing literature suggests that GABA reduces ROS generation, lipid peroxidation, and electrolytic leakage (restoring ion homeostasis), and that it enhances membrane stability. Therefore, there is strong evidence supporting the involvement of GABA in the plant defense system.

One of many potential pathways by which GABA regulates the plant defense system is via modulation of melatonin and JA. GABA is known to enhance the biosynthesis of melatonin in animals, although it is unclear if this occurs directly or indirectly (Kazula et al., 1993). In plants, GABA has been shown to have a synergistic association with melatonin; however, its precise effect on melatonin biosynthesis is not yet known (Lv et al., 2023). Application of melatonin to plants increases defense-related enzyme activity, reduces oxidative stress via antioxidant enzyme activation, and enhances jasmonate content (Liu et al., 2019). Jasmonates, including JA, are important hormone regulators of plant growth and development and are known to enhance resistance against necrotrophs via regulation of defense-related genes (Norman-Setterblad et al., 2000; Fonseca et al., 2009; Sherif et al., 2016).

The effect of exogenous GABA treatment on the stress response induced by WBPH infestation in rice plants has not yet been investigated. Therefore, the present study aimed to quantify GABA induced WBPH resistance in rice plants and evaluate its possible mechanisms. The main focus of the study was to explore the role of GABA in regulation of the antioxidant defense system, TCA cycle enzymes, phytohormone signaling, and water conductance in the setting of WBPH infestation. We hypothesized that exogenous application of GABA induces melatonin biosynthesis, leading to production of JA that regulates expression of pathogenesis-related (PR) genes.

## 2 Materials and methods

### 2.1 Plant material and experimental design

Rice cultivar Ilmi (wild type), obtained from the Plant Molecular Breeding Lab, Kyungpook National University (South Korea) was used as experimental material in this study. The Ilmi rice population was maintained in the Gunwi field, a territory of Kyungpook National University, Daegu, South Korea. All experiments were conducted in the greenhouse in pots. Greenhouse conditions were maintained at 16/8 h dark/light photoperiod, 28°C/26°C temperature, and 60% humidity (Park et al., 2022). The greenhouse used in this study was tent shape, made up of transparent class and the length was 10.2 m, width was 6.6 m, height from side was 2.6 m, and at the middle height was 3.5 m. WBPH, used as herbivorous pest, was provided by the Rural Development Administration of Jeonju, South Korea. The WBPH population was maintained in the insectarium at Kyungpook National University, South Korea. WBPH were kept in separate room in the greenhouse where they were maintained under the same environmental and light conditions as the greenhouse itself. GABA, used as pest stress inhibitor, was obtained from Sigma-Aldrich, (Steinheim, Germany). Uniformly sized seeds of Ilmi rice were soaked in Spotak fungicide (Hankooksamgong, Seoul, South Korea), then placed in an incubator at 33°C for 3 days under dark conditions, as previously described (Kim et al., 2022). The soaked and sprouted seeds were transferred to plastic tray of 50 wholes, (specialized tray for rice growth), and after 30 days, the seedlings were transferred to pots. The plants were grown in specialized soil (Doobaena Plus) provided by Nongkyung Co. Ltd, Korea. The experiment was designed with three biological replicates, each consisting of four groups: control plants, GABA-treated plants, WBPH infested plants, and WBPH infested plants treated with GABA (WBPH+GABA). To identify the optimal GABA concentration, various concentrations (5 mM, 10 mM, and 15 mM) were pre-screened for their effects on seed germination and seedling growth on a plate base. The rice seeds were placed on a three-layered paper within the petri plate, and 5 mL of each GABA concentration in solution were added and then covered the petri plate with lid and grow the seedling for ten days (**Supplementary Figure S1**). Plants were treated with 15 mM GABA (GABA mixed in water and applied as a solution directly as a single treatment) one week before WBPH infestation. The WBPH and WBPH+GABA-treated plants were separately kept in insectarium and infested with100 WBPH per plant.

### 2.2 Histochemical staining and quantitative H_2_O_2_ and O_2_^•-^ assays

*In situ* staining for hydrogen peroxide (H_2_O_2_) was performed by using 3,3-diaminobenzidine (DAB) solution, as described previously (Jan et al., 2021c). Briefly, leaves were excised after one week of infestation, immediately submerged in DAB solution, and incubated for 24 h at 27°C (Chao et al., 2010). The stained leaves were decolorized in boiling ethanol (95% v/v) until brown spots were clearly visualized. After cooling, the leaves were transferred to a solution of lactic acid, phenol, and water (1:1:1, v/v/v) and photographed immediately. For *in situ* superoxide anion (O_2_^•-^) staining, leaves were excised after one week of WBPH infestation, soaked in the trypan blue solution and incubated for 6 h at 25°C. The leaves were de-stained in boiling ethanol (95% v/v), transferred to a solution of lactic acid, phenol, and water (1:1:1, v/v/v) and photographed immediately. For H_2_O_2_ and O_2_^•-^ quantification, excised leaf samples were immediately frozen in liquid nitrogen and stored at −80°C until further use. H_2_O_2_ concentration was measured based on the change in titanium peroxide complex absorbance at 412 nm, as described previously (Willekens et al., 1997). Simultaneously, the O_2_^•-^ generation rate was determined on the basis of nitrite formation from hydroxylamine in the presence of O_2_^•-^ at 530 nm, as described previously (Elstner and Heupel, 1976).

### 2.3 Electrolyte Leakage measurement

Rice leaves were collected after one week of WBPH infestation, and electrolytic leakage was measured as previously described (Khan et al., 2021a). Briefly, fresh rice leaves (0.2 g) were collected after one week of infestation and cut into 5 mm pieces and washed with deionized water to remove surface electrolytes. Thereafter, the samples were kept in a test tube with 10 mL deionized water for 6 h at room temperature. The conductivity of electrolytes (EC1) was measured with a conductivity meter (HURIBA Twin Cond B-173, Japan). The samples were then autoclaved for 15 min at 120°C and cooled to room temperature, at which point electrolyte conductivity (EC2) was measured again. The leakage of ions was calculated using the formula percent electrolytic leakage (EL) = EC1/EC2 × 100.

### 2.4 Relative water and chlorophyll content measurement

To determine the relative water content (RWC), fully mature leaves were randomly collected after one week of infestation and the fresh weight (FW) was measured immediately. Thereafter, the leaves were submerged in distilled water in petri plates for 3 h, to their full turgidity, and the weight was measured again (turgid weight; TW). The same leaves were then dried at 70°C for 48 h and the weight was again measured (dried weight; DW). The relative water content was calculated using the formula RWC (%) = [(FW−DW)/(TW−DW)] × 100.

Chlorophyll content was measured after one month of WBPH infestation using a portable chlorophyll meter (SPAD-502, Konica Minolta, Japan). The second last fully mature leaf was selected for chlorophyll measurement, and readings were taken from the leaf base, middle, and near the tip at the same time. Five leaves were measured from each treatment group, and measurements were averaged to obtain the SPAD value, as described previously (Asif et al., 2022a).

### 2.5 Assays to determine iron, magnesium, and calcium ion accumulation

To evaluate iron (Fe^+^), magnesium (Mg^+^), and calcium (Ca^+2^) ion accumulation, leaf samples were collected after one week of WBPH infestation and immediately lyophilized in freeze drier. About 0.5 g sample was powdered in liquid nitrogen and homogenized in 7 mL 65% nitric acid (HNO_3_) with 1 mL 30% H_2_O_2_, microwaved for 20 min at 180°C, then cooled for 30 min as described previously (Jan et al., 2022). The solvent was further quantified for the presence of the ions by using inductively coupled plasma mass spectrometry (9ICP-MS; Optima 7900DV, Perkin-Elmer, Waltham, MA, USA).

### 2.6 Quantification of ABA and SA

Leaf samples were collected after one week of WBPH infestation and freeze-dried for further use. Dried samples were powdered in liquid nitrogen, and SA and ABA were extracted and quantified by using Sialic Acid (SA) Elisa Kit from LifeSpan BioSciences and Plant Abscisic Acid Elisa Kit from LifeSpan BioSciences, 2401 Fourth Avenue, Suite 900, Seattle. Both the SA and ABA were quantified by using method mentioned in user manual.

### 2.7 RNA isolation and qPCR analysis

Total RNA was extracted from fresh leaves after 12 h of WBPH infestation using an RNeasy Plant Mini Kit (Qiagen, Valencia, CA, USA) following the manufacturer’s instructions. Using RNA as a template, cDNA was synthesized using an UltraScript 2.0 cDNA synthesis Kit following the manufacturer’s instructions. To evaluate selected gene expression levels, qRT-PCR was performed using a qPCRBIO SYBR Green Kit and an Eco Real-Time (Illumina, Singapore) machine. The reaction was performed in 20 µL as previously described (Asif et al., 2022b), and the conditions were as follows: incubation at 95°C for 2 min, followed by 40 cycles at 94°C for 10 s, 60°C for 10 s, and 72°C for 40 s. Actin was used as a reference gene and the reaction was performed in three technical repeats. To validate the actin expression stability, we investigated the expression of actin under all the treatments using three independent biological replicates (**Supplementary Figure S2**). The data was calculated using the ΔΔ^CT^ method.

### 2.8 Free amino acid quantification

Free amino acids were quantified after one week of WBPH infestation. About 500 mg of fresh leaf sample was powdered in liquid nitrogen and homogenized in 70% methanol (10 mL). The homogenate was shaken at room temperature for 24 h. The free amino acid content was determined using an EZ: faast amino acid analysis kit (Phenomex, Santa Clara, CA, USA) following the manufacturer’s instructions. Further, the amino acid content was analyzed by GC-MS using a Hewlett-Packard 6890N/5975 instrument (Agilent Technologies, Torrance, CA, USA) and a ZB-AAA (10 m × 0.25 mm) amino acid analysis column with constant carrier gas flow and an oven temperature program as previously described (Pavlík et al., 2012).

### 2.9 Antioxidant enzyme and lipid peroxidation analysis

For lipid peroxidation and antioxidant enzyme analysis, fresh leaves were collected from each treatment group after one week of WBPH infestation. We used a lipid peroxidation kit from Sigma Korea for the analysis of lipid peroxidation, and the detailed protocol is described in our previous study (Jan et al., 2021a). Meanwhile, ascorbate peroxidase (APX) activity was determined via evaluation of ascorbic acid oxidation as described in detail previously (Imran et al., 2021). 2,2-diphenyl-1-picrylhydrazyl (DPPH), 2,2’-azino-bis(3-ethylbenzothiazoline-6-sulfonic acid (ABTS), chloramphenicol acetyltransferase (CAT), glutathione peroxidase (GPx), superoxide dismutase (SOD), and peroxidase POD activity were evaluated according to the recent protocols (Adhikari et al., 2019; Lubna et al., 2022).

### 2.10 NADPH quantification

Nicotinamide adenine dinucleotide phosphate (NADPH) was quantified using a NADP/NADPH quantification kit from Sigma-Aldrich (Spruce street, St. Louis, USA), following the user manual. Briefly, 50 mg leaf sample was collected after one week of infestation with WBPH and washed with cold PBS (Phosphate-Buffered Saline), then crushed into fine powder in liquid nitrogen. Samples were homogenized in 500 µL NADP/NADPH extraction buffer by freezing and thawing. The homogenate was kept on ice for 10 min, and then centrifuged at 10,000 × g for 10 min. The supernatant (containing extracted NADP/NADPH) was transferred to another tube. Samples were de-proteinized by filtration through a 10 kDa cutoff spin filter. To detect NADP (NADP_total_), approximately 50 µL extracted sample was transferred into a 96-well plate. To detect NADPH, NADP was decomposed by aliquoting 200 µL of extracted sample into microcentrifuge tubes and heating to 60°C for 30 min in a water bath. Samples were cooled and centrifuged; 50 µL of supernatant containing the decomposed sample was then transferred into the 96-well plate. After addition to 100 µL of master reaction mixture (98 µL NADP cyclin buffer and 2 µL NADP cyclin enzyme mix) to each well, the plate was mixed well and incubated at 25°C for 5 min to convert NADP to NADPH. Developer (10 µL) was added into each well and incubated at room temperature for 1 h. To generate NADPH standard, wells with 0, 2, 4, 6, 8, and 10 µL of 10 pmole/µL standard solution were also added to the plate, and the volume of each was brought up to 50 µL with NADP/NADPH extraction buffer. The absorbance was measured at 450 nm (A_450_); readings were taken five times and reactions were run in three technical replicates. The ratio of NADP/NADPH in a sample was determined by the formula ratio = (NADP_total_ – NADPH)/NADPH. The concentration of NADPH was expressed in nmole/mg FW.

### 2.11 Extraction and derivatization of GABA

For GABA extraction, plant samples were collected after one week of WBPH infestation and analyzed as previously described (Weckwerth et al., 2004). GABA was extracted using 300 mg of ground leaves in liquid nitrogen, homogenized in 2 mL chilled solvent containing methanol, chloroform, and water (5:2:1, v/v/v), and stored at −20°C overnight. The homogenate was then shaken for 30 min on ice and centrifuged at 12,000 rpm for 10 min. The supernatant (1.5 mL) was collected carefully and dissolved in 2 mL deionized water and chloroform (2:1). Thereafter, the solution was vortexed vigorously and centrifuged at 12,000 rpm for 2 min. The upper phase was vacuum-dried. GABA was further isolated from the vacuum-dried samples as described previously (Sobolevsky et al., 2003). Briefly, 100 µL acetonitrile and methyl tert-butyldimethylsilyl trifluoroacetamide each were added to each dried sample. Samples were heated for 30 min at 70°C and then 1 µL of each was subjected to gas chromatography (GC Model 7890 A) with BP-5 capillary column. The injector and detector temperatures were kept at 280°C; the oven temperature was maintained at 70°C for 2 min and then increased by 5°C/min to 300°C.

### 2.12 Succinate quantification

Succinate was quantified using Succinate Colorimetric Assay Kit (Sigma-Aldrich, Spruce street, St. Louis, USA), following the manufacturer’s instructions. Briefly, the ground rice leaf tissue (10 mg) from each treatment group was homogenized on ice in succinate assay buffer (100 µL), and then centrifuged at 10,000 × g for 5 min. The supernatant was added directly to a 96-well plate. A final volume of 50 µL per well was maintained by adding succinate assay buffer. Samples from each treatment group were added to 96-well plates in five technical replicates to obtain more accurate results. Wells were mixed by pipetting following addition of 50 µL reaction mix (see **Supplementary Table 1**). The plate was incubated at 37°C for 30 min in dark conditions, and absorbance was then measured at 450 nm (A_450_). Wells were prepared with 0, 2, 4, 6, 8, and 10 µL of 1 nmole/µL succinate standard solution in a total volume of 50 µL succinate assay buffer to generate a standard curve. The absorbance value of the blank well was subtracted from all samples and the succinate concentration (C) was calculated using the following formula: C = (S_a_/S_v_) × 118.09)]

Where:

C is the concentration of final succinate, where S_a_ is the amount of succinate added in the well, S_v_ is the volume of sample in the well, and 118.09 is the molecular weight of succinate.

### 2.13 Statistical analysis

Statistical analysis was performed on all data using GraphPad Prism software (version 5.01; GraphPad, San Diego, CA, USA). The dataset underwent analysis with a one-way analysis of variance (ANOVA) followed by the Bonferroni *post-hoc* test and DMRT. Three independent biological replicates were included in the analysis, and means were subjected to comparison through Bonferroni *post hoc* tests. Significance levels were denoted as follows: *P < 0.05, **P < 0.01, and ***P < 0.001.

## 3 Results

### 3.1 GABA promotes plant growth and reduces WBPH damage

**Figure 1:**
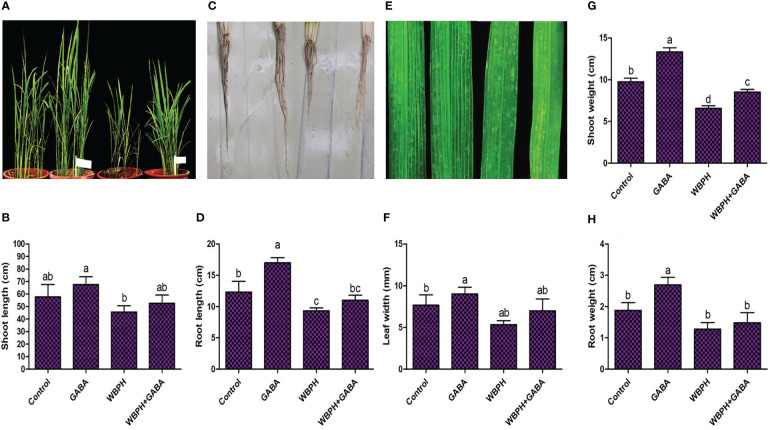
Application of GABA enhance rice plant growth against WBPH stress. The rice seedlings were first treated with GABA and after one week of treatment the plants were infested with WBPH for one month. After one month of infestation, the data presented in this figure was recorded. **(A, B)** shows pictorial and graphical representation of GABA effects on shoot length under WBPH stress. **(C, D)** shows pictorial and graphical representation of GABA effects on root under WBPH stress. **(E, F)** shows pictorial and graphical representation of GABA effects on leaf width under WBPH stress. **(G, H)** shows effect of GABA on shoot and root fresh weight respectively, under WBPH stress. Data represented in graphs were analyzed as a mean of three independent biological replicates ± SD. Different letters on the bars shows significant differences (*p ¾* 0.05) as evaluated by DMRT test.

We investigated the effects of GABA on rice plant growth under normal conditions and when challenged with WBPH infestation. A plate-based study showed that increasing the concentration of GABA significantly (*P* < 0.05) increased germination percentage, as well as the dry and fresh weight of shoots and roots (**Supplementary Figure S1**). The same trend of increased growth and development was also observed after 45 days of GABA supplementation to the pots (**Supplementary Figure S3**). These results show that application of exogenous GABA generally enhances rice plant growth and biomass significantly (*P < 0.05*). Further, we investigated the growth parameters of GABA-supplemented rice plants under WBPH stress after one month (**Figure 1**). GABA significantly (*P* < 0.05) increased the shoot length (15.4%), root length (18.2%), leaf width (32%), and root weight (23.3%) in rice plants under WBPH stress, compared to untreated infested plants. These results indicate that GABA reduces pest stress in rice and promotes plant growth.

### 3.2 GABA inhibits WBPH infestation effects and reduces WBPH population

**Figure 2:**
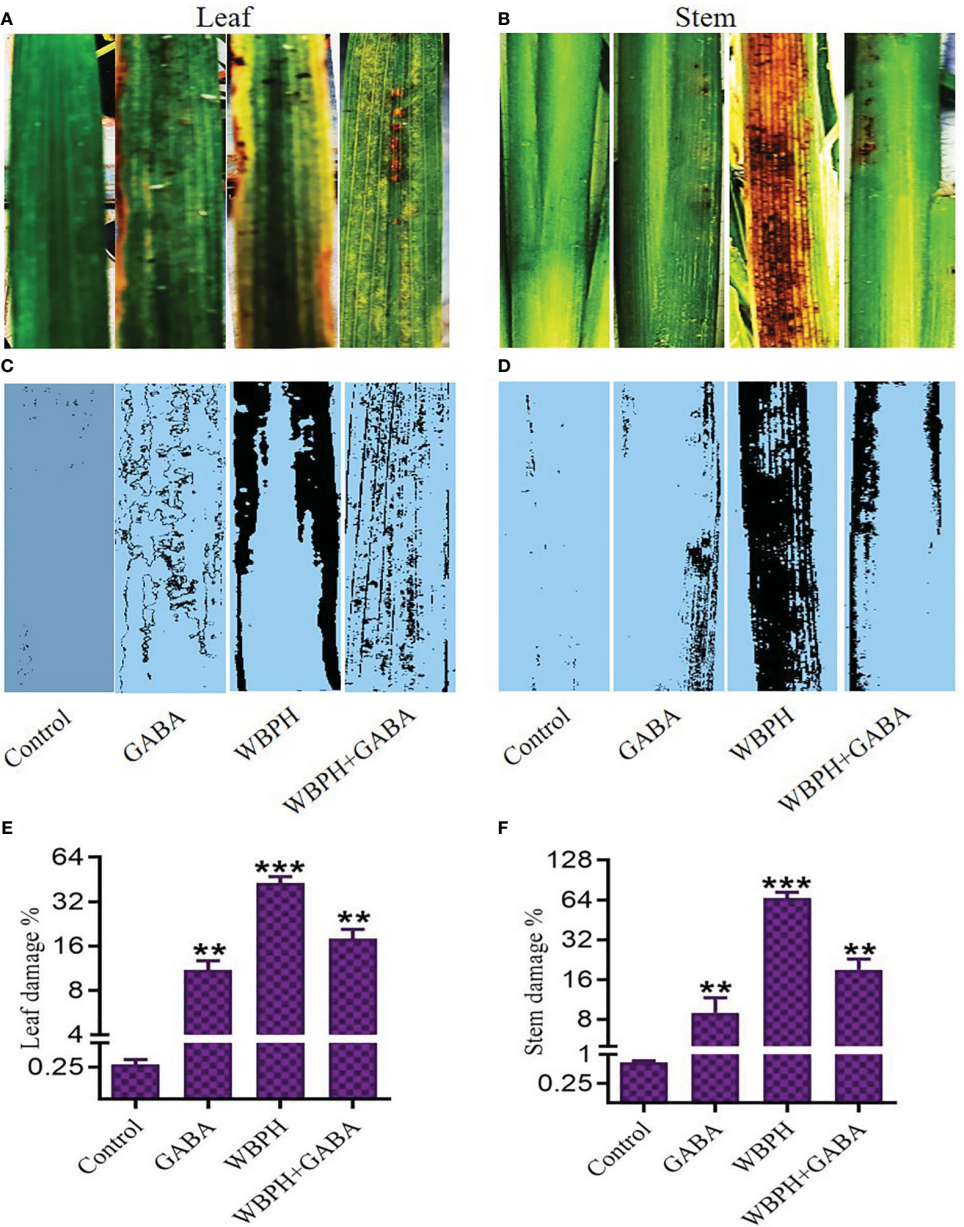
GABA reduces WBPH damagein rice stem and leaves. **(A, B)** shows original picture of leaf and stem respectively. **(C, D)** shows the ImageJ analyzed picture of leaf and stem respectively, indicating the damage induced by WBPH infestation. **(E, F)** shows the quantitative analysis of the leaf and stem damage induced by WBPH, respectively. ** indicates p < 0.01, and *** indicates p < 0.001.

**Figure 3:**
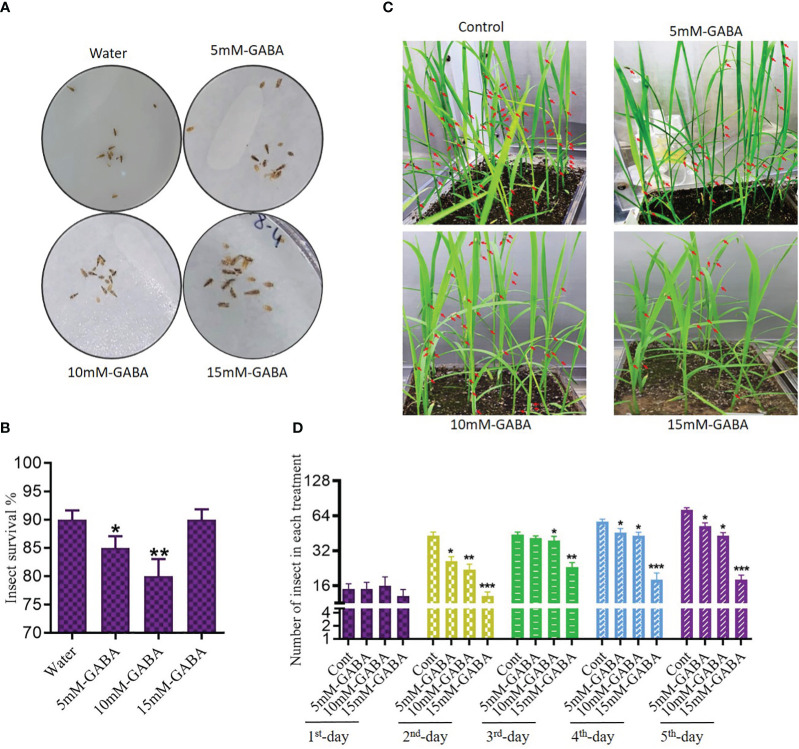
GABA inhibit WBPH survival and reduces their population in rice plant. **(A, B)** shows pictorial and graphical representation of effect of direct application of GABA different concentration on WBPH survival. **(A)** represent dead insects while red arrow in **(C)** indicate presence of WBPH on plants. **(C, D)** shows effect of WBPH population percentage in plants supplemented with different concentrations of GABA. All the control plants, 5mM, 10mM, and 15mM GABA supplemented plants were grown in the same insectarium and were infested seven days after GABA supplementation. DATA in the graphs were presented in percentage. * indicates p < 0.05, ** indicates p < 0.01, and *** indicates p < 0.001.

We next evaluated the spread rate of WBPH infestation in the GABA-treated and non-treated rice plants (**Figure 2**). The plants were infested in the insectarium after one week of supplementation of 5, 10, and 15 mM of GABA. After ten days of infestation, WBPH-damaged leaves and stems of approximately the same size were collected in triplicate, and the damaged area was analyzed by ImageJ software (version 1.8.0). Plants that had been treated with GABA had smaller areas of total damage compared to untreated infested plants. Leaves of non-treated plants exhibited 43% total surface area damage, while this number decreased to 11% in plants treated with 15 mM GABA treatment (**Figure 2A**). Meanwhile, 66% of the stem surface area showed damage in untreated plants, but only 9% of the stem surface was damaged following 15 mM GABA treatment (**Figure 2B**). These results confirmed that GABA supplementation significantly (*P* < 0.05) reduces the effects of WBPH damage in rice plants. To determine the direct effect of GABA on WBPH, 20 insects were subjected to GABA (5, 10, or 15 mM) on plates after two hours of starvation (**Figure 3A**). The GABA was sprayed on tissue paper and put in the plate and then the insects were put into the plate and covered with cotton cloth and the mortality rate was determined after three hours. Dosing the insects directly is not necessarily representative of allowing the insects to feed on plants treated with these concentrations, However, the result showed that WBPH mortality increased with increasing concentrations of GABA (**Figure 3B**), demonstrating that GABA application affects WBPH viability directly. We also evaluated the deterrent effect of GABA on WBPH by applying different concentrations to plants one week prior to WBPH infestation. Approximately 15-20 plants per tray were treated with 5, 10, and 15 mM GABA separately in one insectarium and 150 insect were infested with in the same insectarium and the data related to insect population in each tray of different concentrations of GABA were collected after each day until five days, (**Figure 3C**). Fewer insects were found on plants treated with 15 mM of GABA followed by 10 mM and 5 mM treated plants, than on control plants (**Figure 3D**). The same trend was also observed when plants were infested with WBPH after 45 days of GABA supplementation (**Supplementary Figure S4**). Moreover, we investigated plant recovery rate and rate of infestation spread with different concentrations of GABA supplementation. Overall, we observed that GABA reduced infestation efficiency and induced plant recovery after infestation (**Supplementary Figure S5**). These results indicate that GABA enhances the plant defense system in response to pest stress from WBPH.

### 3.3 GABA reduces oxidative stress and regulates ion homeostasis during WBPH infestation

**Figure 4:**
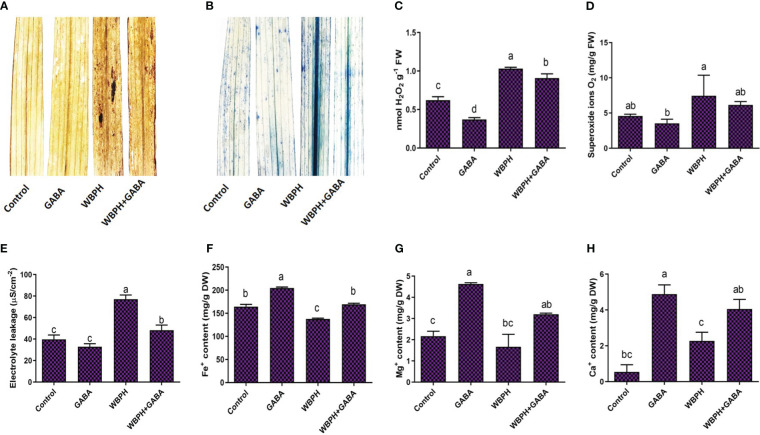
Application of GABA reduces oxidative stress, induced by WBPH infestation in rice plants and regulate ions homeostasis. **(A, B)** shows *in situ* detection of oxidative stress caused by generation of ROS during WBPH stress, using DAB and trypan blue staining respectively. **(C-E)** shows H_2_O_2_, O_2_^.−^, and electrolytic leakage. **(F-H)** shows Fe^2+^, Mg^+^, and Ca^+^ contents. Data represented in graphs were analyzed as a mean of three independent biological replicates ± SD. Different letters on the bars shows significant differences (*p ¾* 0.05) as evaluated by DMRT test.

To further evaluate the effect of GABA treatment on rice plants under WBPH stress, we investigated the level of oxidative stress induced by insect infestation (**Figure 4**). Oxidative stress occurs due to generation of ROS such as H_2_O_2_ and O_2_^•-^ under stress conditions. We quantified H_2_O_2_ and O_2_^•-^ production by visualizing it via DAB and trypan blue staining and observed that WBPH infestation increased their accumulation; this effect was mitigated in the GABA-treated plants (**Figures 4A, B**). Quantitative analysis of H_2_O_2_ and O_2_ **^•^** ^-^ also revealed that WBPH infested plants showed significant accumulation of both ROS species (*P* < 0.05 compared to un-infested plants), whereas GABA supplementation reduced H_2_O_2_ by 12% and O_2_ **^•^** ^-^ by 17% (*P* < 0.05; **Figures 4C, D**). These results indicate that GABA application significantly mitigates oxidative stress induced by WBPH via reduction of ROS generation.

The generation of ROS in plants is typically accompanied by electrolyte leakage and programmed cell death. We observed that electrolyte leakage also reduced by 37% in WBPH+GABA plants compared with WBPH infested plants (**Figure 4E**), which strongly suggest that electrolytic leakage and ROS are correlated. We extended our investigation to determine changes in accumulation of Fe^+^, Mg^+^, and Ca^+^ ions with GABA supplementation in response to WBPH stress (**Figures 4F–H**). GABA application significantly (*P* < 0.05) increased the total concentration of Fe^+^, Mg^+^, and Ca^+^ ions by 237, 100, and 23respectively compared with WBPH infested plants. Interestingly, Ca^+^ in WBPH treated plants increased 317% compared with control plants, which shows that WBPH also induces Ca^+^ accumulation in rice plants. These results suggested that GABA regulates ion homeostasis under WBPH stress in rice plants. Furthermore, compared to control plants, GABA-treated plants also showed reduced ROS and electrolyte leakage in normal condition, while enhancing Fe^+^, Mg^+^, and Ca^+^ ions accumulation. These results suggested that in normal situation, GABA reduces ROS and induces ions (Fe^+^, Mg^+^, and Ca^+^) accumulation in rice plants.

### 3.4 GABA regulates succinate and NADPH levels via shunt pathway genes under WBPH stress

**Figure 5:**
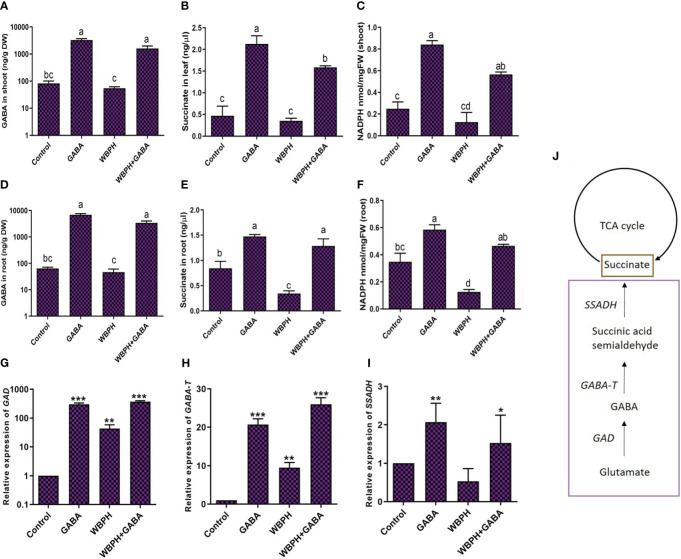
Exogenous application of GABA regulate TCA cycle in rice plant. **(A, B)** shows accumulation of GABA in rice shoot and roots respectively. **(C, D)** shows accumulation of succinate in leaf and root respectively. **(E, F)** shows accumulation of NADPH in shoot and root respectively. **(G-I)** shows the expression of GABA shunt genes i.e GAD, GABA-T, and SSADH respectively. **(J)** shows schematic representation of GABA shunt and TCA cycle pathway. Data represented in graphs were analyzed as a mean of three independent biological replicates ± SD. Different letters and on the bars shows significant differences (*p ¾* 0.05) as evaluated by DMRT test. Asterisks on bars shown in **(G-I)** represent significant differences (*p ¾* 0.05) as evaluated by Bonferroni *post-hoc* test.

We next evaluated levels of endogenous GABA, succinate, NADPH, and GABA shunt pathway genes (*GAD*, *GABA-T*, *SSADH*) in rice plants in response to challenge with WBPH (**Figure 5**). We first quantified endogenous GABA content in rice roots and shoots following WBPH infestation (**Figures 5A, B**). Our results showed that a significantly higher amount of GABA accumulated in roots and shoots in the GABA-supplemented plants (GABA, GABA+WBPH) compared with control plants. However, GABA accumulation in the shoots reduced by 34% in WBPH infested plants compared with control plants and by 51% in GABA+WBPH plants compared with un-infested GABA-supplemented plants (**Figure 5A**). The same trend was found in the roots, whereas GABA was reduced by 27% in WBPH infested plants compared to control plants and by 51% in GABA+WBPH plants compared with un-infested GABA-supplemented plants (**Figure 5B**). The concentration of succinate and NADPH in both the roots and shoots of rice plants followed the same trend, decreasing in response to WBPH infestation but increasing with GABA treatment (**Figures 5C–F**). Succinate concentration increased 356% in GABA-treated plant leaf compared with control plant and 354% in WBPH+GABA plant leaf compared with WBPH infested plants (**Figure 5C**). In the roots, the increase in succinate concentration with GABA treatment was more pronounced in infested plants (275%) than un-infested plants (74%; **Figure 5D**). NADPH activity was also enhanced by GABA application and reduced by WBPH infestation in both shoots and roots (**Figures 5E, F**).

GABA shunt is mainly composed of three enzymes: GAD, GABA-T, and SSDHA (Bouché et al., 2003). We next evaluated the expression of genes in the GABA shunt pathway following GABA supplementation and WBPH infestation in rice plants. We found that the expression of *GAD* increased significantly (88%; *P* < 0.05) by GABA treatment following WBPH infestation (**Figure 5G**). The same trend was seen with expression of *GABA-T* and *SSADH* (**Figures 5H, I**). The expression of *GABA-T* enhanced 172% in GABA+WBPH plants compared with untreated WBPH infested plants (**Figure 5H**). Meanwhile, expression of SSADH was significantly enhanced (107%) in GABA-supplemented plants compared with controls, and 16% increased with GABA treatment in infested plants compared with control plants (**Figure 5I**). Together, these results reveal that application of GABA reduces WBPH stress via regulation of succinate concentration, NADPH activity, and shunt pathway gene expression.

### 3.5 GABA induces pathogen defense-related genes and ABA and SA hormones

**Figure 6:**
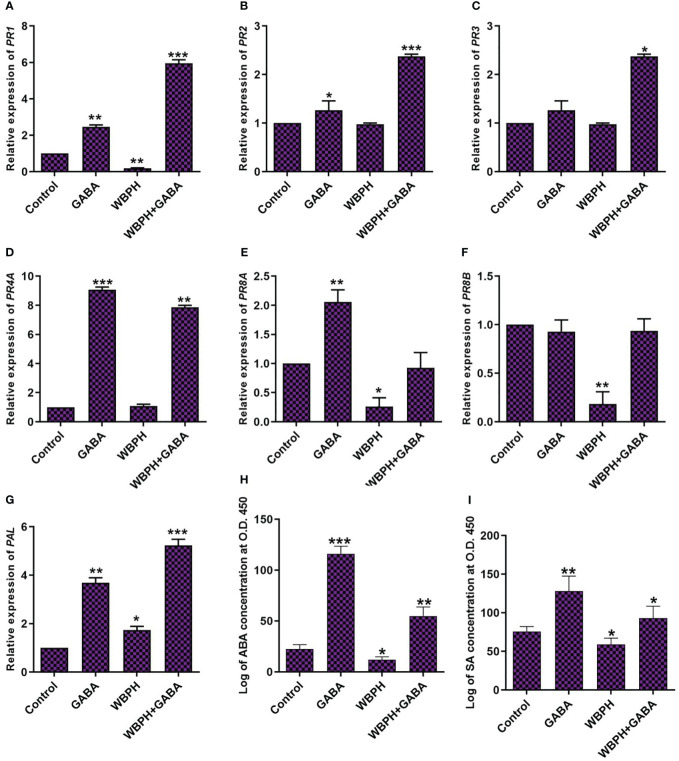
GABA induces Phenylalanine ammonia-lyase, pathogenesis related genes and phytohormons in rice under WBPH stress. **(A)** represent *PAL* gene expression level. **(B-G)** represent gene expression of *PR1*, *PR2*, *PR3*, *PR4A*, *PR8A*, and *PR8B* respectively. **(H, I)** shows the accumulation of ABA and SA hormones respectively. Data represented in graphs were analyzed as a mean of three independent biological replicates ± SD. Asterisks on bars shown in **(G-I)** represent significant differences (*p ¾* 0.05) as evaluated by Bonferroni *post-hoc* test.

In most plants, pathogenesis-related (PR) genes accumulate in response to pathogen infection and are used as marker genes for systemic acquired resistance. Here, we evaluated the expression of phenylalanine ammonia-lyase (*PAL*) and some PR genes, together with ABA and SA (**Figure 6**). We investigated the expression pattern of *PAL*, *PR1*, *PR2*, *PR3*, *PR4A*, *PR8A*, and *PR8B* in GABA-supplemented rice plants challenged with WBPH stress. The results showed that, although WBPH infestation decreased expression of *PR1* and *PR8A*, GABA treatment ultimately increased the expression of *PAL*, *PR1*, *PR2*, *PR3*, and *PR4A* (*P < 0.05*) during WBPH-induced stress (**Figure 6**). We also found that GABA induces *PAL*, *PR1*, *PR2*, *PR4A*, and *PR8A* expression in normal conditions, without pest infestation. However, GABA had no apparent effect on *PR8A* and *PR8B* in the setting of WBPH stress (**Figures 6F, G**). Overall, these results show that GABA application significantly regulates certain PR genes in response to WBPH-induced stress.

ABA and SA are stress hormones that accumulate under both abiotic and biotic stress to provide protection (Bharath et al., 2021; Nadarajah et al., 2021). Here, we investigated the trend of SA and ABA accumulation in GABA-treated plants during WBPH-induced stress (**Figures 6H, I**). We found that exogenous GABA enhances ABA and SA accumulation in normal conditions as well as during WBPH-induced stress. Under normal conditions, GABA increased ABA and SA accumulation by 413% and 69%, respectively. In WBPH infested plants, GABA treatment induced respective ABA and SA increases of 352% and 57%. Overall, our results indicate that GABA has a more pronounced effect on ABA accumulation.

**Figure 7:**
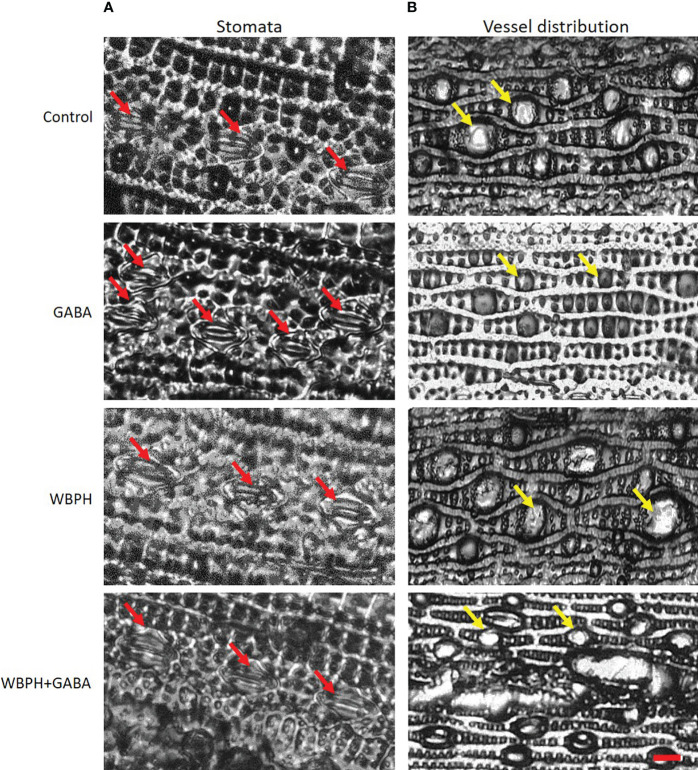
Exogenous application of GABA regulates rice leaf stomata closure and leaf vessels reduction. **(A)** shows stomata opening and closure and **(B)** shows vessels dimeter variations. Red arrows shows stomata while yellow arrows shows vessels in leaf lamina.

Maintenance of water content via stomata control and leaf vessel distribution is an important component of the plant stress response. Our analysis demonstrated that GABA application regulates stomata opening and closing and water use efficiency of the plants. The results showed that GABA inhibit stomata opening in GABA and GABA+WBPH treated plants (**Figure 7A**), which is in line with ABA accumulation (**Figure 6H**) as ABA induces stomata closing. Further, we predicted that GABA application could regulate water use efficiency due to reducing leaf vessel cell size. As expected, we found that GABA application reduced leaf vessel size under both normal and WBPH infestation conditions (**Figure 7B**). It has been reported previously that smaller vessel cell size reduces water conductivity, resulting in less water evaporation (Apgaua et al., 2017). Overall, these results indicate that GABA application induces ABA accumulation, ultimately resulting in water conservation that helps to promote stress resistance.

### 3.6 GABA reduces WBPH-mediated oxidative stress via regulation of the antioxidant system

**Figure 8:**
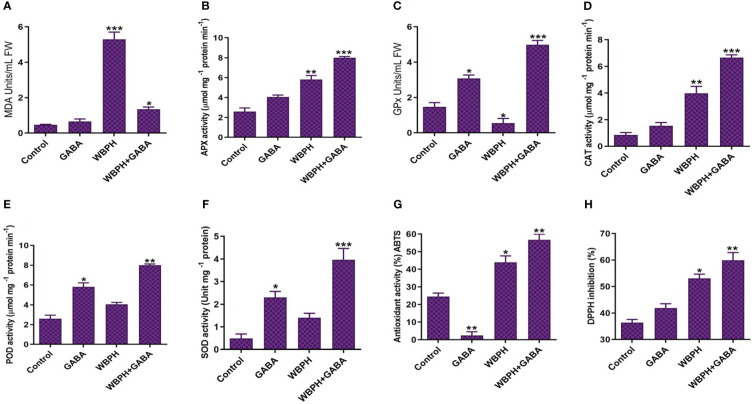
GABA application induces antioxidant defense system in rice plant against WBPH stress. **(A)** shows MDA contents, **(B)** shows APX activity, **(C)** shows GPx activity, **(D)** shows CAT activit, **(E)** shows POD activity, **(F)** shows SOD activity, **(G)** shows ABTS activity, and **(H)** shows DPPH activity. Data represented in graphs were analyzed as a mean of three independent biological replicates ± SD. Asterisks on bars shown in **(G–H)** represent significant differences (*p ¾* 0.05) as evaluated by Bonferroni *post-hoc* test.

To understand the mechanism of WBPH-induced stress inhibition by GABA application in rice plants, we observed the activity of antioxidant enzymes. First, we used lipid peroxidation, measured by malondialdehyde (MDA) content, to assess membrane damage induced by WBPH infestation (**Figure 8A**). Our results revealed that GABA application reduced MDA content by 291% in WBPH+GABA plants compared WBPH infested plants. In addition, we investigated the activity of antioxidant enzymes (APX), glutathione peroxidase (GPx), CAT, POD, SOD, ABTS, and DPPH. All of these increased significantly (*P* < 0.05) in the GABA+WBPH plants compared with untreated, un-infested control plants. The activities of GPx, POD, and SOD increased significantly (*P* < 0.05) by the application of GABA in both normal and WBPH infested plants, suggesting that exogenous application of GABA alone upregulates the activity of these enzymes. Furthermore, our results showed that ABTS and DPPH activity was significantly higher in WBPH and GABA+WBPH plants compared with control plants. However, the activity of both the enzymes increased by 29% and 12%, respectively, in GABA+WBPH plants than that of WBPH plants. In this study we also found that in normal condition, GABA application significantly (*P* < 0.05) reduces ABTS activity (about 90%) compared with control plants, whereas in WBPH stress and WBPH stress supplemented with GABA showed enhance ABTS activity (**Figure 8G**). Our results suggest that GABA reduces ABTS activity in normal conditions, but increases its activity when plants undergo stress.

### 3.7 GABA induces melatonin biosynthesis genes in response to pest stress

**Figure 9:**
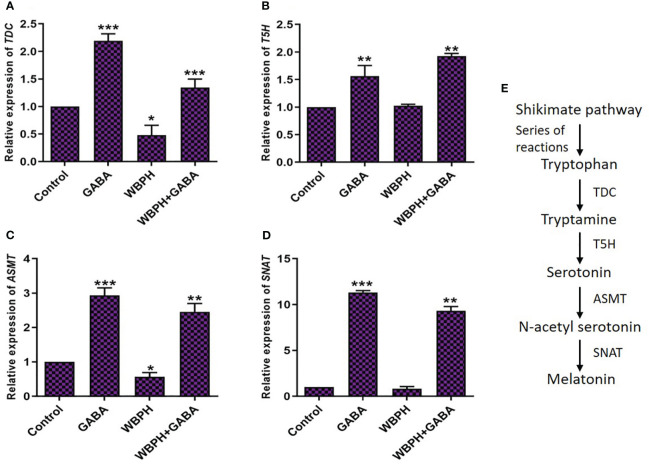
GABA induces melatonin biosynthesis in response to WBPH stress. **(A–D)** shows the transcript level of melatonin biosynthesis pathway genes i.e. *TDC*, *T5H*, *ASMT*, and *SNAT* respectively. **(E)** shows the general pathway of melatonin and their genes. Data represented in graphs were analyzed as a mean of three independent biological replicates ± SD. Asterisks on bars shown in **(A–D)** represent significant differences (*p ¾* 0.05) as evaluated by Bonferroni *post-hoc* test.

GABA and melatonin are plant endogenous molecule that are synergistically associated to take part in regulation of plant responses to stress conditions. Therefore, we next evaluated the effect of exogenous GABA application on expression of melatonin biosynthesis genes tryptophan decarboxylase (*TDC*), tryptamine 5-hydroxylase (*T5H*), acetyl-serotonin methyltransferase (*ASMT*), and serotonin N-acetyl transferase (*SNAT*) in response to WBPH-induced stress. Generally, *TDC* and *T5H* regulate the production of serotonin from tryptophan, and *ASMT* and *SNAT* produce melatonin from serotonin (Bhowal et al., 2021). **Figure 9C** shows the melatonin biosynthesis pathway in plants. We studied differential expression of all the four genes and found that all transcript levels were significantly expressed in the plants supplemented with GABA (**Figure 9**). *TDC* and *ASMT* were significantly (*P* < 0.05) reduced, by 52% and 43%, respectively, in WBPH infested plants compared with control plants (**Figures 9A, C**). However, GABA application in WBPH infested plants increased their respective transcript levels by 186% and 337%. These results indicate that exogenous GABA application rescues and overcompensates for decreased transcription of *TDC* and *ASMT* caused by WBPH infestation. Similarly, GABA application significantly increased the expression of *T5H* and *SNAT* in the setting of WBPH infestation compared with normal WBPH infested plants (**Figures 9B, D**). As all four genes related to melatonin biosynthesis are significantly upregulated by GABA in the setting of WBPH stress, enhancement of melatonin accumulation by GABA may represent a potential stress protection mechanism.

### 3.8 GABA regulates JA pathway genes under pest stress

**Figure 10:**
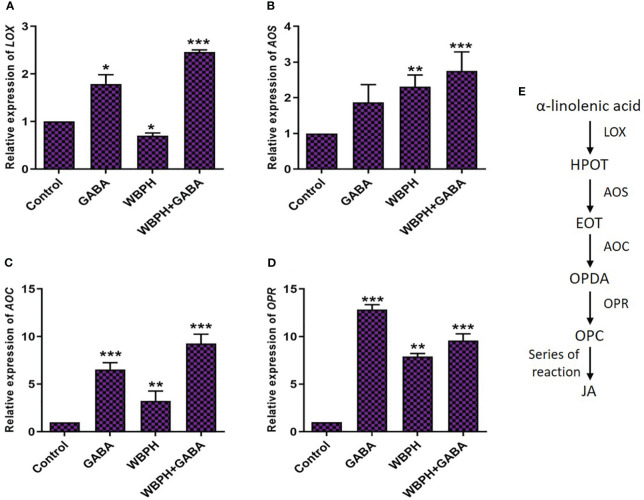
GABA induces JA biosynthesis pathway in response to WBPH stress. **(A–D)** shows the transcript level of JA biosynthesis pathway genes i.e. *LOX*, *AOS*, *AOC*, and *OPR* respectively. **(E)** shows the general pathway of JA and their genes. Data represented in graphs were analyzed as a mean of three independent biological replicates ± SD. Asterisks on bars shown in **(G–I)** represent significant differences (*p ¾* 0.05) as evaluated by Bonferroni *post-hoc* test.

The transcript levels of genes related to the JA biosynthesis pathway (*LOX*, *AOS*, *AOC*, *OPR*) were assessed in WBPH infested plants (**Figure 10**). *LOX* is a marker gene for JA biosynthesis pathway and express against pest infestation (Shrestha and Huang, 2022). *AOS* and *AOC* play a key role in transforming the product generated by *LOX*, specifically hydroperoxyoctadecatrienoic acid (HPOT). This process leads to the creation of the intermediate known as 12-oxo-phytodienoic acid (OPDA). This intermediate (OPDA) exhibits independent signaling activity (Taki et al., 2005). Nevertheless, in the process of synthesizing jasmonic acid (JA), it undergoes transportation from the chloroplast to the peroxisomes. Within the peroxisomes, *OPR* facilitates its reduction, followed by a series of steps involving β-oxidation to shorten the side chain (Shrestha and Huang, 2022). The ultimate outcome within the peroxisomes is JA, and it has the ability to move freely into the cytosol. **Figure 10E** shows the general pathway of JA biosynthesis in plants. The expression levels of all four genes showed significant (*P* < 0.05) increases in the GABA-supplemented, WBPH infested plants compared with untreated, un-infested control plants. Expression levels of *LOX*, *AOS*, *AOC*, and *OPR* were increased in response to WBPH stress in untreated plants. With GABA treatment, *LOX*, *AOS*, *AOC*, and *OPR* expression further increased by 78%, 17%, 187%, and 21%, respectively. Overall, these results indicate that *LOX*, *AOS*, *AOC*, and *OPR* genes are transcribed in response to WBPH stress, leading to JA accumulation, and that the upregulation is enhanced by treatment with exogenous GABA.

### 3.9 GABA application reduces WBPH stress via regulation of free amino acid biosynthesis

Amino acids play a crucial role in pest–plant interaction. They are important components of plant primary metabolites and function as precursors for the synthesis of important metabolites. To quantify changes in the free amino acid content of GABA-supplemented rice plants undergoing WBPH stress, leaf samples were collected after one week of WBPH infestation and amino acids were quantified through GC-MS. WBPH infestation and GABA supplementation both significantly (*P* < 0.05) affected the free amino acid content (**Supplementary Figure 6**). GABA application to non-infested plants increased the concentration of most amino acids. However, WBPH infested plants demonstrated overall reduced free amino acid content compared to control plants, even when treated with GABA. Compared to control, infested plants and GABA-treated plants showed an increased accumulation of free amino acids. Specifically, GABA treatment increased aspartic acid, alanine, arginine, and proline by 11%, 8%, 24%, and 12%, respectively, compared to control plants. However, WBPH infestation significantly (*P* < 0.05) reduced the accumulation of aspartic acid, alanine, arginine, proline and total amino acid approximately to 30%, 34%, 23%, 26%, and 62% respectively compared to control plants. Comparing free amino acids of WBPH infested and WBPH+GABA plants, most of them were significantly enhanced in WBPH+GABA plants. In particular, aspartic acid, glutamic acid, alanine, leucine, arginine, proline and total amino acids were increased approximately to 24%, 18%, 25%, 27%, 27%, 31%, and 99% respectively, in WBPH+GABA plant compared to WBPH infested plants. This result revealed that these free amino acids are inhibited by WBPH stress, whereas they are induced by GABA to reduce the WBPH-induced stress in rice plants.

## 4 Discussion

**Figure 11:**
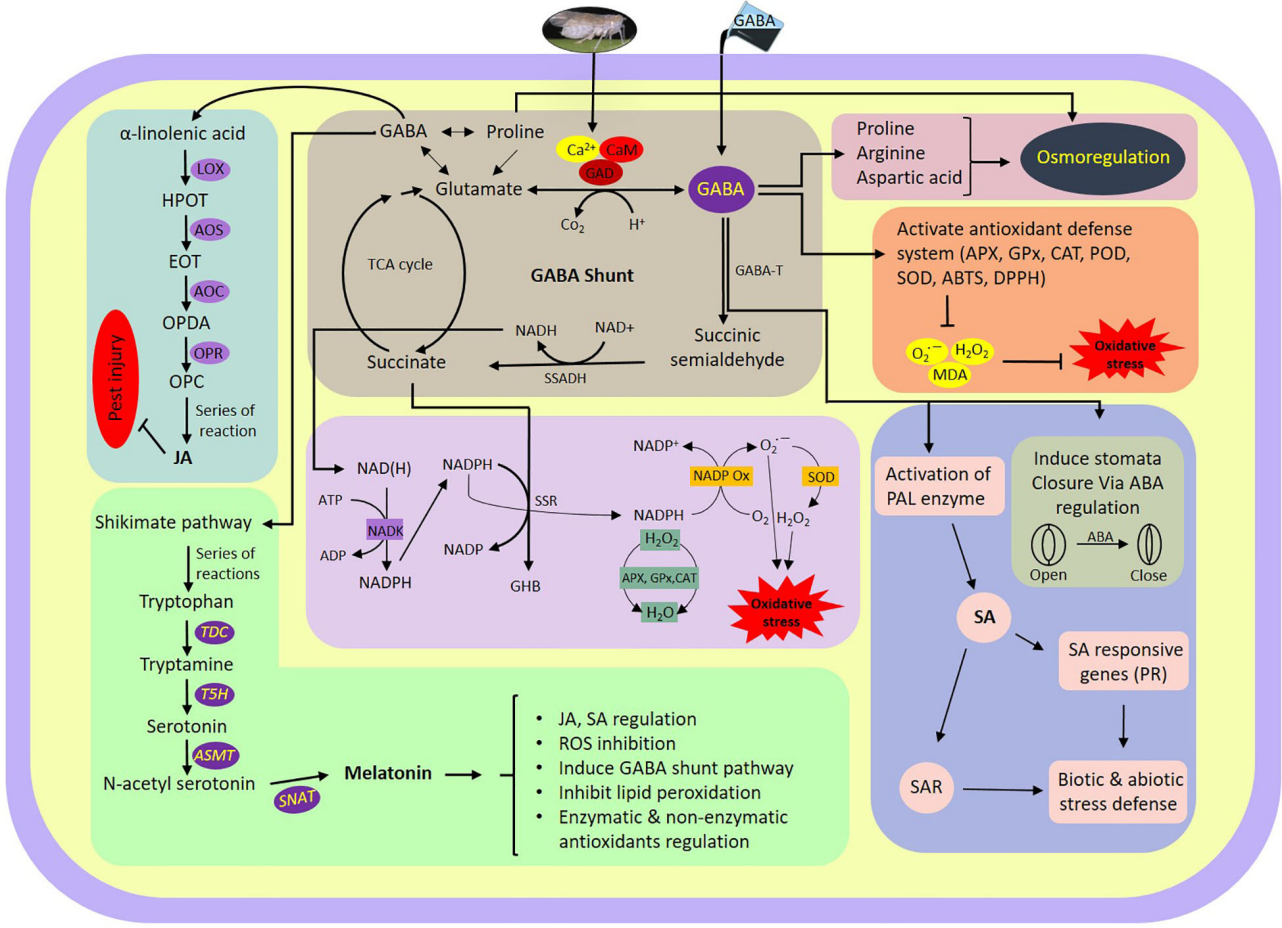
Schematic representation of GABA shunt and its associated pathways regulated during WBPH stress in rice plant. ABA, Adenosine triphosphate; ABTS, Azino-bis(3-ethylbenzothiazoline-6-sulfonic acid; ADP, Adenin di-phosphate; AOC, Allene oxide cyclase; AOS, Allene oxide synthase; APX, Ascorbate peroxidase; ASMT, Acetyl-serotonin methyltransferase; ATP, Adenin tri-phosphate; Ca2+, Calcium Ion; CaM, Calmodulin; CAT, Ahloramphenicol acetyltransferase; DPPH, 2,2-diphenyl-1-picrylhydrazyl; EOT, Epoxyoctadecatrienoic acid; GABA, Gamma-aminobutyric acid; GABA-T, GABA transaminase; GAD, Glutamate decarboxylase; GHB, γ-Hydroxybutyric acid; GPx, Glutathione peroxidase; H_2_O_2_, Hydrogen peroxide; HPOT, Hydroperoxyoctadecatrienoic acid; JA, Jasmonic acid; LOX, Lipoxygenase; MDA, Malondialdehyde; NAD+, Nicotinamide adenine dinucleotide; NADP+, Nicotinamide adenine dinucleotide phosphate; NADH, Nicotinamide adenine dinucleotide+hydrogen; NADK, NAD+ kinase; NADPH, Nicotinamide adenine dinucleotide phosphate+hydrogen; NADPOX, NADPH oxidase; O_2_^•−^, Superoxide radical; OPC, Oxo-phytodienoic acid; OPDA, Oxophytodienoic acid; OPR, Oxo-phytodienoic acid reductases; PAL, Phenylalanine ammonia-lyase; POD, Peroxidase; PR, Pathogenesis related; ROS, Reactive oxygen species; SAR, Systemic acquired resistance; SNAT, Serotonin N-acetyl transferase; SOD, Superoxide dismutase; SSADH, Semialdehyde dehydrogenase; SSR, Succinic semialdehyde reductase; T5H, Tryptamine 5-hydroxylase; TDC, Tryptophan decarboxylase.

The data presented here demonstrate a possible mechanism by which GABA regulates plant tolerance against WBPH infestation (simplified model presented in **Figure 11**). We found that GABA regulates several stress tolerance pathways, including antioxidant enzyme activity, the TCA cycle, phytohormones, melatonin biosynthesis-related genes, JA biosynthesis-related genes, ion concentration, stomata opening and closing, and water conductance.

GABA is known as a metabolic signaling amino acid that accumulates in response to biotic and abiotic stress in plants [35,53,54]. To the best of our knowledge, no other study has explored the potential of exogenous GABA application to enhance the tolerance of rice against WBPH. The plate and pot-based results of this study show that increasing concentrations of GABA significantly improved rice seedling growth parameters compared to control plants (**Supplementary Figure S1**, **Supplementary Figure S3**). It also enhanced rice growth when applied exogenously during WBPH infestation, which normally inhibits plant growth (**Figure 1**). However, an overall decline in growth was still observed in GABA+WBPH plants compared to control plants, indicating an inability for GABA application to fully rescue plants from infestation. Plants produce very low levels of GABA at baseline; however, its production increases promptly when plants are subjected to stress (Kinnersley and Turano, 2000). It has been reported that GABA promotes tomato growth via regulation of photosynthetic machinery, gas exchange capacity, chlorophyll biosynthesis, enzymatic and non-enzymatic responses, and membrane stability during stress conditions (Luo et al., 2011). Our previous and current studies demonstrated that WBPH infestation has a severe effect on rice plant growth attributes (Jan et al., 2021b). This investigation showed that exogenous GABA reduces the area of WBPH-induced damage, enhances plant recovery rate after WBPH attack, and discourages WBPH infestation of rice plants (**Figures 2**, **3**; **Supplementary Figure S5**). Presumably, GABA application either increases the insect deterrent effects of the plant or directly affects insects. Our results are supported by previous studies reporting that GABA acts as a key factor in the plant defense against herbivorous insects, and that insects feeding on GABA show a reduced performance (Scholz et al., 2017). Additionally, a GABA-supplemented diet reduced the growth and survival of another herbivorous insect, *Choristoneura rosaceana* cv Harris, and delayed its life cycle progression; while its attack on soybean leaves increased GABA accumulation (Ramputh and Bown, 1996). A similar study reported recently that *Spodoptera littoralis* larvae fed with a GABA-supplemented diet showed reduced weight compared to control larvae groups, whereas their infested plants showed increased levels of GABA accumulation (Scholz et al., 2015). The feeding of *S. littoralis* larvae on *Arabidopsis* and mechanical wounding of the plant with a robotic caterpillar both increased endogenous GABA, by two and ten-fold, respectively (MithoüFer et al., 2005; Scholz et al., 2015). Another study reported that GABA production was induced locally in tobacco and soybean leaves when challenged with insect damage (Bown et al., 2002). In support of previous studies, our results reveal that GABA is involved in the plant defense system against herbivorous insects.

In this study, we found that exogenous GABA treatment induced endogenous GABA accumulation in both roots and shoots. Non-treated plants infested with WBPH also showed enhanced levels of GABA accumulation compared to control plants, showing that WBPH attack induces a GABA biosynthesis pathway. GABA is mainly produced by decarboxylation of glutamate, catalyzed by GAD, and degraded by GABA-T in plants (Shelp et al., 2012b). *GAD* and *GABA-T* expression levels were highest in WBPH+GABA plants compared to WBPH infested plants; simultaneously, both the genes were also significantly upregulated in WBPH infested plants compared to controls (**Figure 5**). *GAD* activity is Ca^2+^ dependent and is induced by plant cell injury. We found that Ca^2+^ contents were increased after the non-treated plants were infested with WBPH and WBPH+GABA plants showed more Ca^2+^ contents than the WBPH infested normal plants (**Figure 4**), which shows that Ca^2+^ and GABA has synergistic association. It is reported that GAD is activated in one of two ways: (i) in intact plant tissue and neutral pH; GAD activity is stimulated in a Ca^2+^-dependent manner by the binding of calmodulin (CAM) to the CAM-binding site, or (ii) after wounding of plant cells, the vacuolar content is released and the cytosol is acidified, leading to a Ca^2+^-independent activation of GAD (Carroll et al., 1994; Snedden et al., 1995). Our results are at par with previous studies, suggesting an elevation of Ca^2+^ under herbivorous attack in *Arabidopsis* (Dengler, 2006; Kiep et al., 2015). Therefore, it is likely that without GABA application, wounding of rice plant leaves also induces Ca^2+^ upregulation sufficient for GAD activation, ultimately resulting in GABA accumulation.

The application of GABA prior to WBPH infestation limited plant cell death and generation of ROS such as H_2_O_2_ and O_2_ **^•^** ^-^ (**Figure 4**). Here, we demonstrated that GABA application significantly overcame the oxidative stress induced by WBPH, as MDA content was greatly reduced in GABA+WBPH plants compared to untreated plants infested with WBPH (**Figure 8A**). The simultaneous increase of antioxidant enzymes (APX, GPx, CAT, POD, SOD, ABTS, and DPPH) (**Figure 8**) in the GABA+WBPH plants suggested that GABA stimulates the antioxidant system and reduces the generation of ROS during WBPH stress. To date, there are no known observations reported showing that GABA treatment induces antioxidant enzymes against WBPH in rice plants. However, recent studies demonstrated that tomato and pear plants treated with exogenous GABA prior to pathogenic fungus infection showed increased antioxidant enzyme activity and reduced oxidative stress (Yu et al., 2014; Fu et al., 2017; Yang et al., 2017). Recently, it was reported that exogenous GABA increases CAT and SOD activity, which results in a significant reduction in H_2_O_2_, O_2_ **^•^** ^-^, and MDA content in response to heavy metal stress (Seifikalhor et al., 2020). Reduction of ROS by exogenous GABA is likely one of the main strategies used to overcome the oxidative stress induced by WBPH infestation. Here, our finding of ROS reduction was paralleled by increased antioxidant enzyme activity after GABA application. Additionally, our study revealed that the GABA shunt reactions were activated in response to WBPH stress in rice plants. The expression levels of *GAD*, *GABA-T*, and *SSADH* were upregulated in WBPH+GABA plants compared to only WBPH infested plants while only GABA application alone also led to an increased level of all three genes (**Figure 5**). These results were in line with a previously reported study reporting that exogenous GABA increased the expression of *GABA-T* and *SSADH* in citrus plants (Hijaz and Killiny, 2019). Furthermore, our study found higher accumulation of NADPH and succinate in GABA+WBPH plants compared to only WBPH infested plants, while WBPH infested plants reduced NADPH and succinate accumulation when compared with control plants (**Figure 5**). However, we found that succinate was reduced in WBPH infested plants compared with control plants; however, the expression level of *SSADH* was higher in WBPH infested plants compared to the control plants. There was non-significant increase in SSADH expression level in WBPH infested-plants compared to control plants. SSADH provide succinate into TCA cycle by conversion of succinate semialdehyde (SSA) into succinate (Shelp et al., 2021). Succinate provides a carbon skeleton and NADH through the TCA cycle, which produces ATP through an electron transport chain, which prevents the accumulation of ROS (Tuin and Shelp, 1994; Fait et al., 2005; Hijaz and Killiny, 2019). Here, we infer that exogenous application of GABA promotes GABA shunt activation during stress conditions, maintaining an adequate ATP supply and reducing ROS generation.

Notably, the accumulation of free amino acids, particularly proline, arginine, aspartic acid, and glutamic acid, was inhibited by WBPH stress but increased by GABA supplementation (**Supplementary Figure S6**). These amino acids collectively reduces oxidative stress in rice plant by enhancing various biochemical and physiological processes. Proline is an important osmo-protectant that accumulates in response to various stressors (Che-Othman et al., 2020). Being an ROS scavenger and an influencer of ion homeostasis, proline interacts with hydroxyl radicals and is considered a GABA precursor (Cuin and Shabala, 2005; Kaul et al., 2008; Signorelli et al., 2015). In addition to proline, there was accumulation of arginine and glutamic acid, which are glutamate derivatives; their accumulation was consistent with the induction of GABA shunt. The interaction between proline and the NADP+/NADPH ratio has also been reported in several studies (Kohl et al., 1988; Hare and Cress, 1997; Strizhov et al., 1997). Proline oxidizes NADPH and is reduced by H_2_O_2_ for redox regulation and prevention of H_2_O_2_ toxicity (Seifikalhor et al., 2020). The accumulation of free amino acids induced by exogenous GABA is likely involved in the control of oxidative stress.

In-depth characterization of the effects of exogenous GABA treatment can allow for a better understanding of WBPH stress inhibition mechanisms in rice plant. Regarding gene expression and phytohormonal regulation induced by GABA, we found that *PAL* expression, PR gene expression, and accumulation of ABA and SA were significantly increased in GABA-supplemented plants (in both infested with WBPH and non-infested plants) (**Figure 6**). Recent reports are in line with our findings, demonstrating that GABA treatment induces *PAL* and *PR1* gene expression significantly in plants (Abd Elbar et al., 2021; Mejri et al., 2023). PAL is an important enzyme of the phenylpropanoid pathway that catalyzes the transition of phenylalanine into precursors of phenolic compounds such as flavonoids, lignins, and SA (La Camera et al., 2004; Vogt, 2010). Furthermore, our investigation showed that expression of JA biosynthesis-related genes (*LOX*, *AOS*, *AOC*, and *OPR*) were significantly induced in GABA-treated plants infested with WBPH (**Figure 10**), indicating both SA and JA are important signals involved in the GABA-mediated WBPH defense response. Research has shown that the SA and JA pathways can have additive or synergistic effects, particularly in rice plants infested by pests. However our results shows that SA and JA some time act in complementary manner. For instance SA and JA pathways was observed complementary in rice infested with WBPH (Kanno et al., 2012). The induction of PR genes by GABA, under the regulation of SA, might be part of a broader defense mechanism that is also effective against insect pests like WBPH. This could be due to the complex interactions between the SA and jasmonic acid (JA) pathways, which are not exclusively antagonistic but can sometimes work together to enhance the plant’s resistance to various stressors. The significance lies in the potential for these PR genes to contribute to a defense response that is relevant to the specific stress caused by WBPH, despite the traditional association of SA with pathogen defense. We further hypothesized another possible mechanism of GABA-mediated defense against WBPH, via regulation of melatonin. We found that melatonin biosynthesis-related genes (*TDC*, *T5H*, *ASMT*, and *SNAT*) were significantly induced by GABA in both infested and non-infested plants (**Figure 9**). There are two possible ways that the melatonin biosynthesis pathway is being induced in this case: either GABA induces melatonin biosynthesis-related genes directly, or it causes accumulation of JA that leads to melatonin production. This inference is based on a previous study, which showed that GABA and melatonin have a synergistic function in response to multiple types of stress (Shomali et al., 2021). It has also been reported that methyl jasmonate induces melatonin biosynthesis in watermelon (Li et al., 2021a). Additionally, melatonin treatment increases methyl jasmonate via induction of *LOX* and *AOC* expression, resulting in regulation of antioxidant enzymes and reduction of H_2_O_2_ (Liu et al., 2019). These reports show that melatonin and methyl jasmonate are part of a positive feedback loop, and work synergistically in the stress response. Considering these previous studies, our results suggest that melatonin synthesis, mediated by GABA and JA, inhibits WBPH -induced stress in rice.

GABA exhibits interactions with phytohormones such as auxins, cytokinins, abscisic acid, and ethylene, suggesting a potential contribution to stress tolerance in plants (Podlešáková et al., 2019). It is reported that, IAA and ABA induced aluminum-activated malate transporter (*ALMT*) family genes which is responsible for GABA regulation under stress condition (Podlešáková et al., 2019). Another study demonstrated the functional link between GABA and cytokinins in barley, where transgenic lines overexpressing the *Arabidopsis* cytokinin dehydrogenase 1 gene (*AtCKX1*) resulted in the upregulation of GABA-related genes *GAD* and *ALMT* in roots (Pospíšilová et al., 2016). Exogenous application of GABA also increased ethylene by regulation of 1-aminocyclopropane-1-carboxylic acid (ACC) synthase which is an evidence of GABA and ethylene interaction (Kathiresan et al., 1997). Interestingly, GABA application enhanced the accumulation of ABA in both normal and infested plants which regulate stomata closing, and reduced water conductance via leaf vessel size inhibition (**Figure 6**, 7). The closing of stomata is consistent with ABA accumulation, as ABA induces stomata opening during stress conditions to reduce water loss. We infer that GABA induces ABA accumulation, which results in closing of stomata and reduced water conductance under stress conditions. There is clear evidence from a previous study that GABA application plays a key role in stomata closure (Mekonnen et al., 2016). Another study focused on drought stress also demonstrated that ABA accumulation induces Ca^2+^ flux and the Ca^2+^/calmodulin complex further activates GAD, resulting in GABA synthesis (Shelp et al., 2012a; Daszkowska-Golec and Szarejko, 2013). The *GAD1* mutant of *Arabidopsis* shows higher transpiration even in drought stress conditions, but ABA supplementation leads to closing of stomata and reduced water loss (Ward and Schroeder, 1994; Meyer et al., 2010). This result supports our finding that GABA plays a pivotal role in stomata closure and water conductance via induction of ABA accumulation. In plants, the accumulation of γ-aminobutyric acid (GABA) is a rapid response to environmental stress, and changes in its internal levels impact plant growth. Applying GABA externally has been found to enhance stress tolerance by influencing the expression of genes related to plant signaling, transcriptional regulation, hormone production, reactive oxygen species generation, and polyamine metabolism.

Undoubtedly, the primary benefit of enhancing the GABA content in crops and food matrices lies in its significant potential to positively impact human health, and consumers are already acquiring tomatoes with genetically enhanced GABA (Ahmar et al., 2023a, Ahmar et al, 2023b). However, there are pros and cons of increasing dietary intake of GABA in long term and it is predicted that it might prevent and alleviate high blood pressure effects (Gramazio et al., 2020). In term of plants, it is reported that high accumulation of GABA has the potential to disrupt the balance of amino acids within cells, resulting in abnormal phenotypes (Gramazio et al., 2020). Recently, it is reported that transgenic tomato increased GABA accumulation up to 19 fold but the plants scarcely produced fruits, with some exhibiting teratogenic effects, displayed pronounced dwarfism, pale green, and curled compound leaves, along with necrosis on both leaves and buds (Lee et al., 2018). In the study conducted by (Koike et al., 2013), the successful elevation of GABA levels was achieved through the suppression of *SlGABA-T1* using RNA interference. However, the resultant transgenic plants exhibited dwarf phenotypes, with heights less than half of the wild type (WT), and infertility, accompanied by significant flower abscission. Similarly, the GABA-T-deficient mutant *pop2* in *Arabidopsis* demonstrated deficiencies in pollen tube growth, as well as impaired cell elongation in hypocotyls and primary roots, as observed in studies by (Palanivelu et al., 2003; Renault et al., 2011). According to our knowledge, there is insufficient information exists on the effect of long-term use of GABA on agricultural. It is crucial to investigate whether continuous or extended use of GABA may lead to unintended consequences, such as altered plant physiology, changes in soil microbial communities, or potential ecological impacts.

## 5 Conclusion

The current study concludes that WBPH infestation significantly inhibits the growth and development of rice plants, while exogenous GABA application increased WBPH stress tolerance and enhanced rice growth. GABA application either has a direct effect on WBPH, or is involved in triggering a downstream defense reaction in the infested plants. It is evident from our study that GABA mitigated WBPH stress via regulation of the TCA cycle, which results in reduction of ROS generated by pest infestation. Another possible mechanism of GABA-mediated WBPH stress reduction in rice is associated with *PAL* gene regulation, which is responsible for activation of PR genes and SA hormone biosynthesis. Moreover, GABA induced antioxidant-related enzyme activity, which increased ROS scavenging and reduced WBPH-induced stress. Our study also suggests that GABA enhances ABA accumulation, which results in stomata closure and reduction of water loss. Additionally, GABA reduces oxidative stress via enhanced accumulation of melatonin and upregulates JA biosynthesis pathway, which is a defense signaling pathway in pathogen resistance. Overall, our study found that GABA inhibits WBPH stress in rice plants through regulation of several defensive mechanisms, which merit further molecular evaluation.

## Data availability statement

The original contributions presented in the study are included in the article/**Supplementary Material**. Further inquiries can be directed to the corresponding author/s.

## Author contributions

RJ: Conceptualization, Investigation, Writing – original draft, Writing – review & editing. SAsi: Formal analysis, Software, Writing – review & editing. SAsa: Conceptualization, Methodology, Writing – original draft. L: Formal analysis, Writing – review & editing. ZK: Formal analysis, Writing – review & editing. WK: Formal analysis, Writing – review & editing. K-MK: Supervision, Validation, Visualization, Writing – original draft.
