# Supplementary material for: Gamma-aminobutyric acid treatment promotes resistance against Sogatella furcifera in rice
Source: Front Plant Sci. 2024 Jul 18;15:1419999. doi: 10.3389/fpls.2024.1419999 (PMC11291254; doi:10.3389/fpls.2024.1419999)
Supplement: Supplementary file 1 [file DataSheet_1.docx]

Supplementary table 1. Reaction mixes

| **Reagent** | **Sample and Standards** | **Sample blank** |
| --- | --- | --- |
| Succinate assay buffer | 42 µL | 44 µL |
| Succinate converter | 2 µL | ---- |
| Succinate enzyme mix | 2 µL | 2 µL |
| Succinate substrate mix | 2 µL | 2 µL |
| Succinate developer | 2 µL | 2 µL |


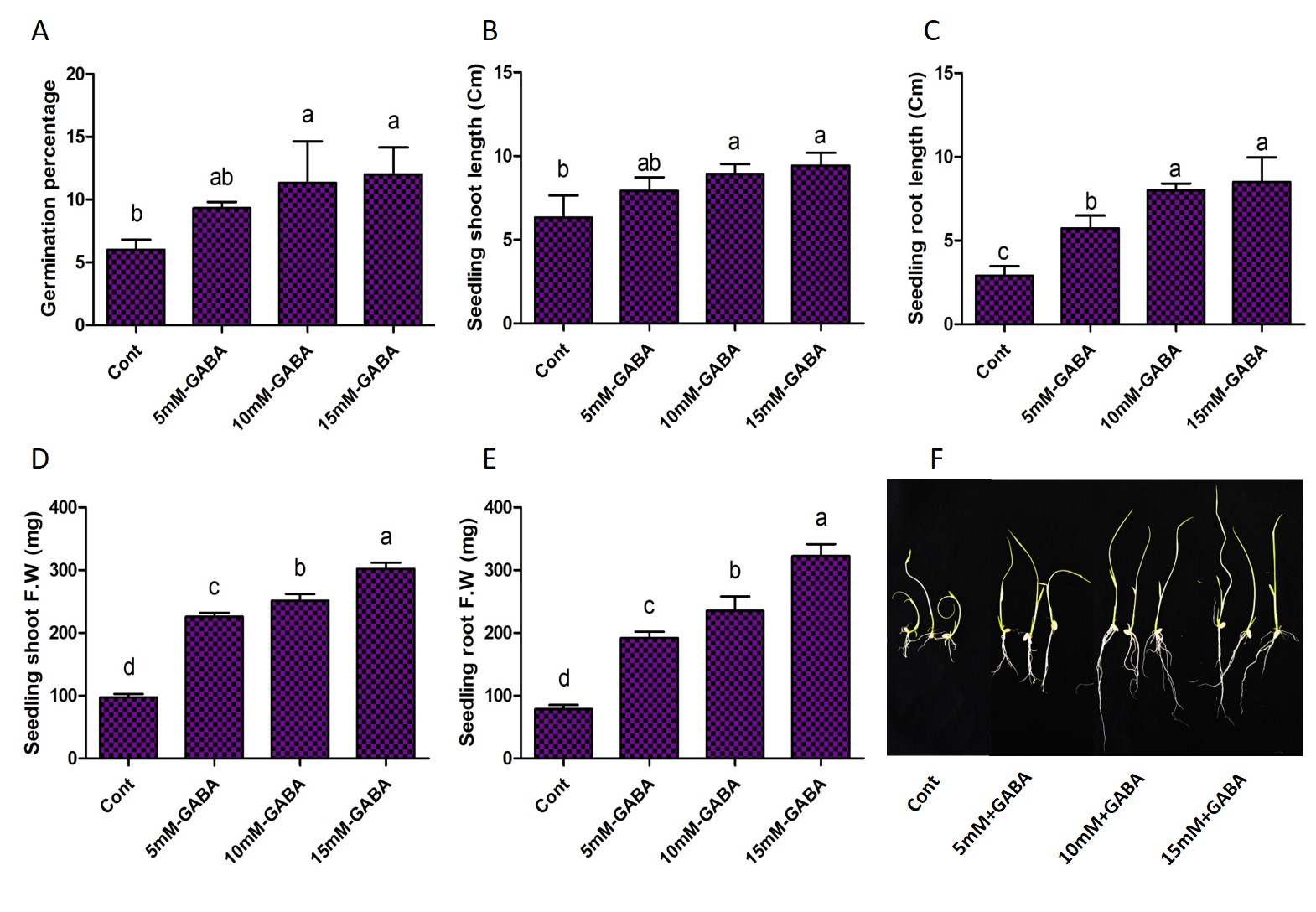


**Supplementary Fig. S1.** Plate-based evaluation of different concentrations of GABA on rice germination percentage, root and shoot length, and root and shoot fresh weight. (A) shows the rice seed germination percentage under different concentrations of GABA. (B and C) show shoot length and root length, respectively. (D and E) show shoot and root fresh weight, respectively, and (F) represent a pictorial representation of rice seedling root and shoot length. Data represented in graphs were analyzed as a mean of three independent biological replicates ± SD. Different letters and on the bars show significant differences (*p <* 0.05) as evaluated by DMR test.


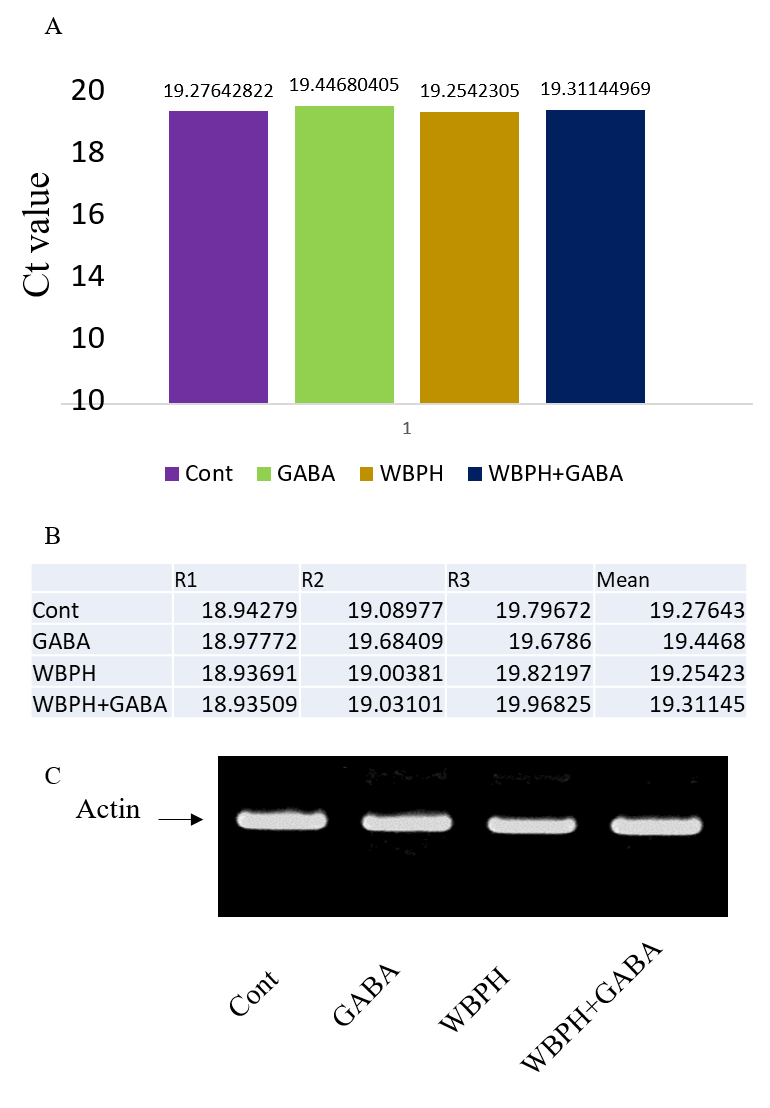


**Supplementary Fig. S2.** Validation of actin stable expression in all the treated and control plants. (A) shows the graph of Ct value of actin under all the treatments. The data represented in the graph is the mean of three independent biological replicates. (B) is the tabulated representation of the Ct value of actin where R1, R2, and R3 shows the value of each biological replica. (C) shows the bands of actin bands of PCR product.


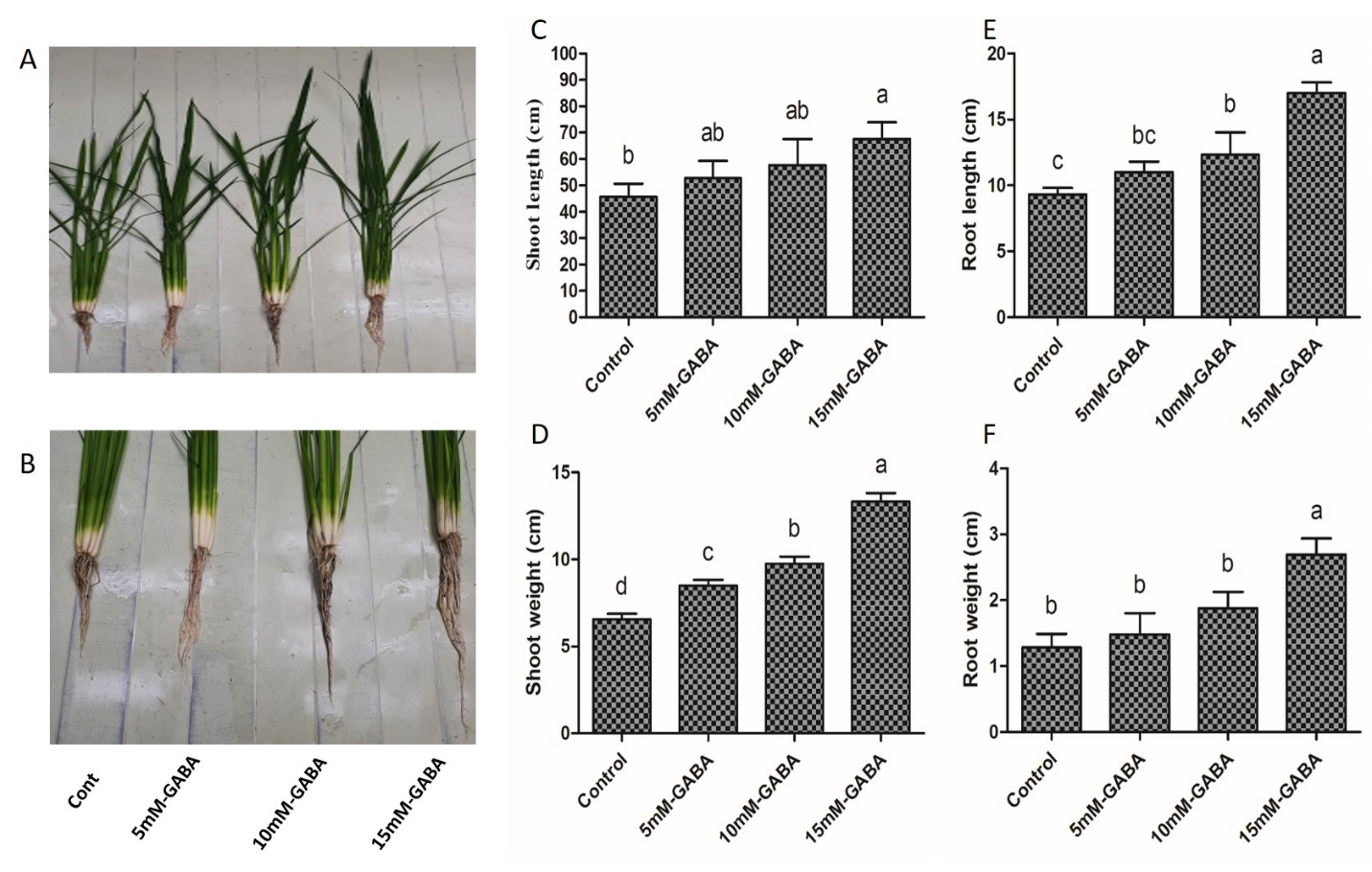


**Supplementary Fig. S3.** Pot base analysis of GABA effect on rice root shoot length and root shoot fresh weight. (A and B) shows pictorial representation of shoot length and root length respectively, supplemented with different concentrations of GABA. (C and D) shows graphical representation of shoot length and shoot fresh weight respectively, and (E and F) shows root length and root fresh weight respectively. Data represented in graphs were analyzed as a mean of three independent biological replicates ± SD. Different letters and on the bars shows significant differences (*p <* 0.05) as evaluated by DMRT test.


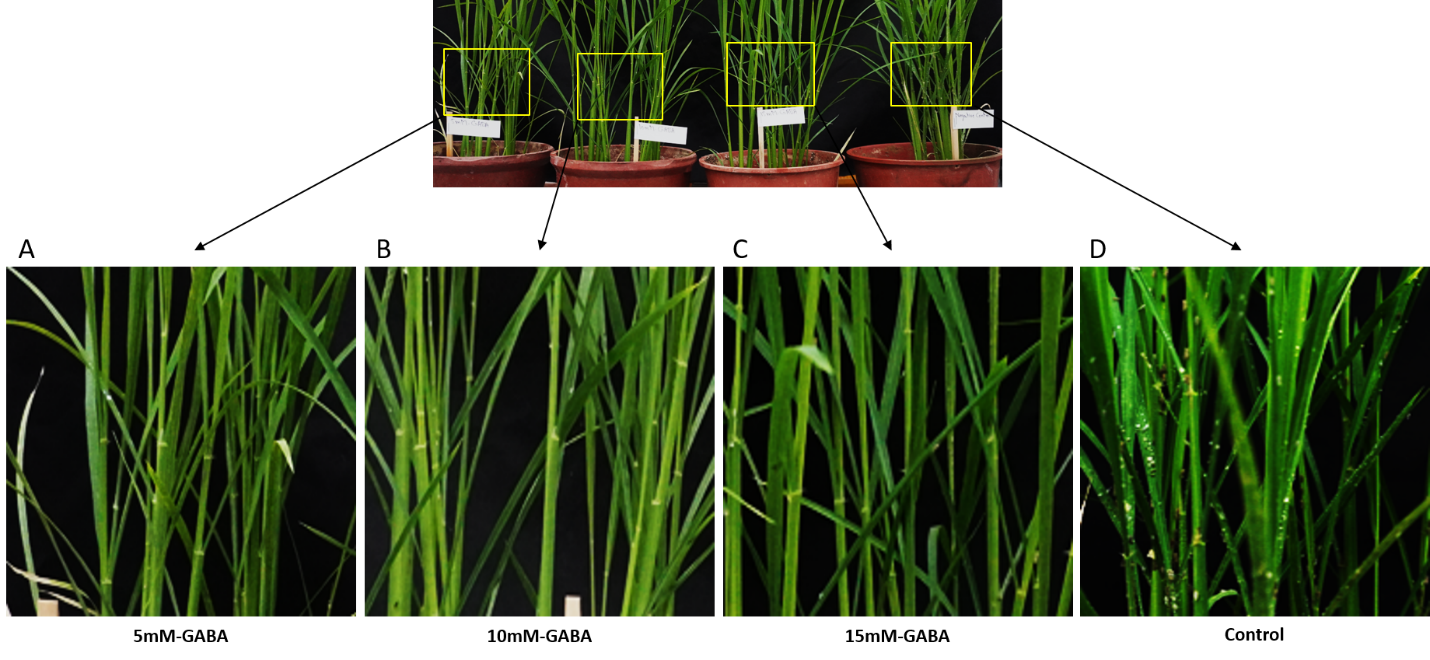


**Supplementary Fig. S4.** Evaluation of GABA different concentrations effect on WBPH population in rice plant.


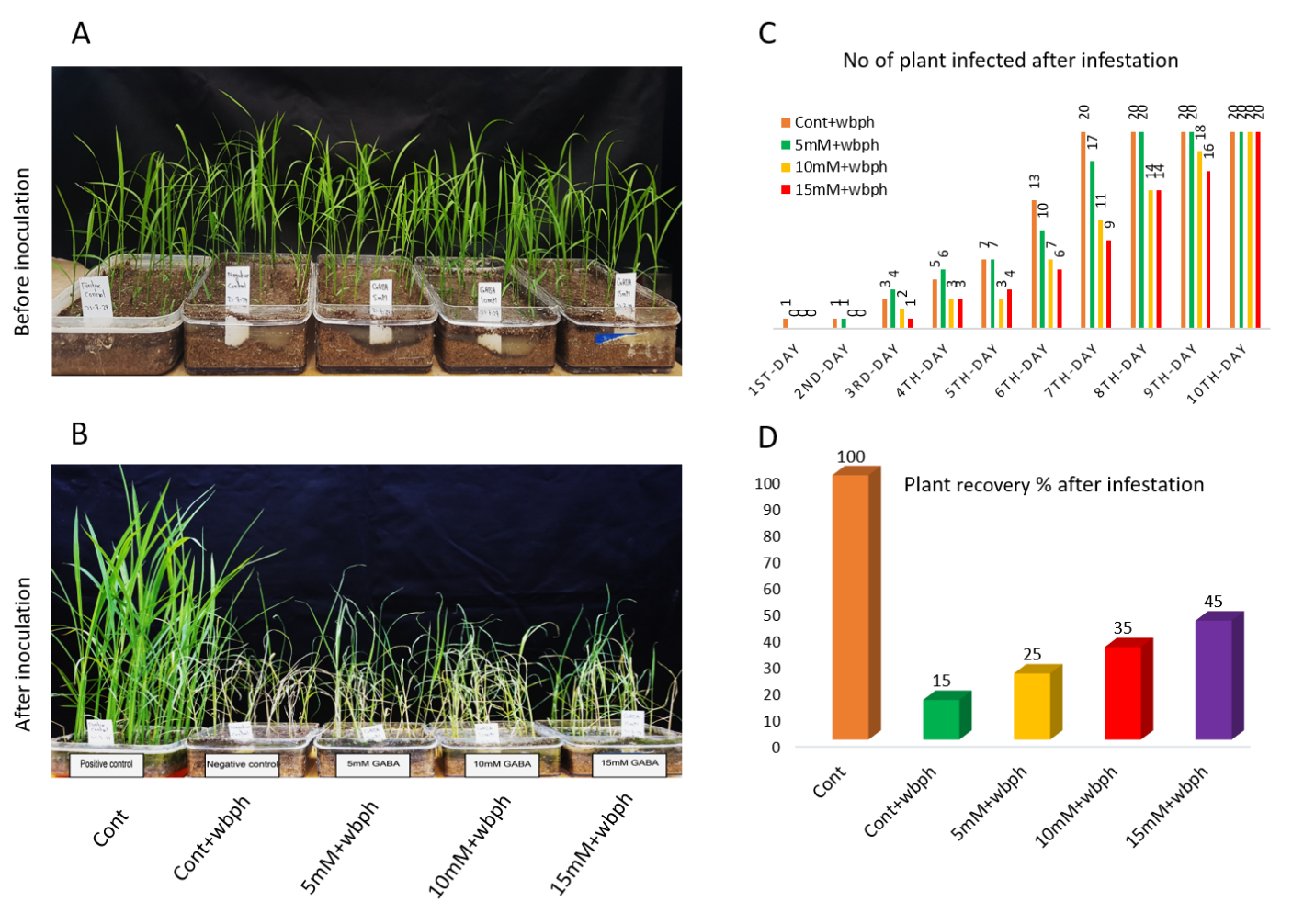


**Supplementary Fig. S5.** Determination of plant recovery rate after WBPH infestation, enhanced by GABA application. (A) shows rice seedling before GABA and WBPH infestation. (B) plants after WBPH infestation and application of GABA different concentration after seven days of infestation. (C) shows plants number infected with each day interval until ten days. (D) shows the plant recovery rate after WBPH damage and GABA supplementation. Data represented in graphs were analyzed as a mean of three independent biological replicates ± SD. Different letters and on the bars shows significant differences (*p <* 0.05) as evaluated by DMRT test.

**
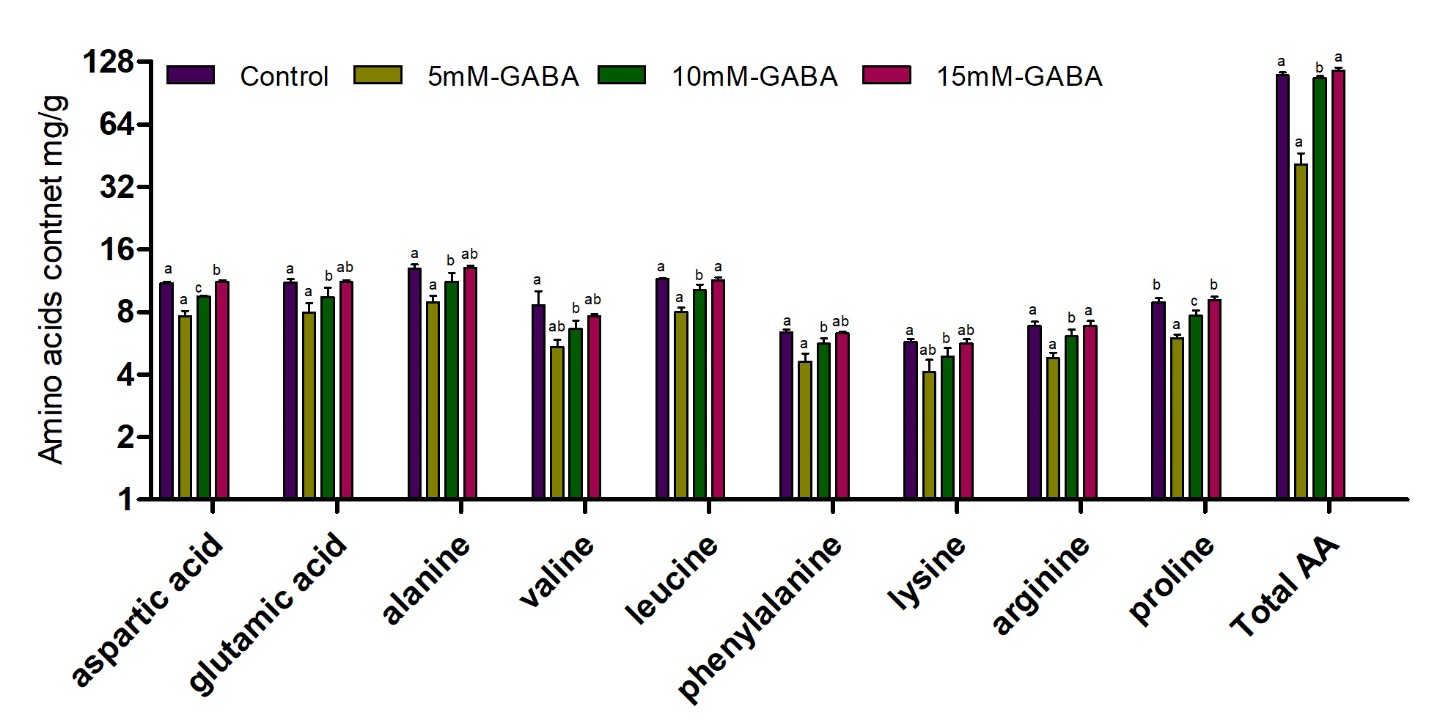
**

**Supplementary Fig. S6.** Free amino acid contents regulation under WBPH stress and GABA application. Data represented in graphs were analyzed as a mean of three independent biological replicates ± SD. Bars labeled with different letters are significantly different (p < 0.05) as evaluated by DMRT analysis.
